# 3D Printing and Electrospinning of PLLA-*co*-CL/PDLA Blends as Potential Materials for Cardiovascular Implants

**DOI:** 10.1021/acsbiomaterials.6c00151

**Published:** 2026-04-28

**Authors:** Hanin Alkhamis, Angelika Ritschel, Lennard K. Shopperly, Sahar Salehi, Axel T. Neffe, Katarzyna Polak-Kraśna

**Affiliations:** † Institute of Active Polymers, 305196 Helmholtz-Zentrum Hereon, Kantstraße 55, 14513 Teltow, Germany; ‡ Institute of Functional Materials for Sustainability, Helmholtz-Zentrum Hereon, Kantstraße 55, 14513 Teltow, Germany; § Charité—Universitätsmedizin Berlin, Corporate Member of Freie Universität Berlin and Humboldt-Universität zu Berlin, Centre for Trauma- and Reconstructive Surgery, Hindenburgdamm 30, 12203 Berlin, Germany; ∥ Professor for Engineering Biointelligent System, Institute of Food Science and Biotechnology, University of Hohenheim, Garbenstrasse 25, 70599 Stuttgart, Germany; ⊥ Fraunhofer Institute for Manufacturing Engineering and Automation (IPA), Nobelstrasse 12, 70569 Stuttgart, Germany; # Institute of Materials Chemistry, BTU Cottbus-Senftenberg, Universitätsplatz 1, 01968 Senftenberg, Germany

**Keywords:** Stereocomplexation, Biodegradable Polymers, PLLA-*co*-CL, PDLA Electrospinning, 3D Printing, Cardiovascular Biomaterials, Cardiac Coverings, Endothelialization

## Abstract

Cardiovascular implants are limited by the lack of materials that combine mechanical strength with bioactivity to support endothelial function, prevent thrombosis, and promote long-term integration. Conventional polymers often lack these biological features, underscoring the need for multifunctional biomaterials. This study evaluates poly­[(l-lactide)-*co*-(ε-caprolactone)]/poly­(d-lactide) (PLLA-*co*-CL/PDLA) blends processed via 3D printing and electrospinning and assesses their suitability as cardiovascular implant coverings through material characterization and endothelial cell interactions. Blend compositions were fabricated as films and meshes, and optimized constructs were selected for cytocompatibility and endothelial biofunctionality testing. Endothelial responses were evaluated over 7 days through viability assays and quantification of vasoactive mediators, including Thromboxane B_2_ (TXB_2_), Prostacyclin (PGI_2_), and Nitric Oxide (NO). Among all formulations, the 90:10 PLLA-*co*-CL/PDLA blend exhibited the most favorable wettability (76.5° ± 4.0), indicating enhanced hydrophilicity at intermediate compositions. Electrospun meshes supported high HUVEC viability (>80%) and metabolic activity, while 90:10 films maintained ∼67% viability after 7 days. Functional analysis showed increasing PGI_2_ release over time, reaching ∼966 pg/mL on 90:10 films, whereas TXB_2_ rose moderately. The PGI_2_/TXB_2_ ratio shifted from pro-thrombotic (<1) on day 1 to antithrombotic (>1) by day 7, indicating endothelial stabilization. NO release increased to ∼0.82 μmol/L on films and ∼0.9 μmol/L on meshes, reflecting improved endothelial function and vascular homeostasis. Hemolytic potential was assessed via red blood cell lysis assays. All films and meshes exhibited negligible hemolysis (0.027–0.660%), comparable to a silicone elastomer reference (0.153%) and substantially lower than slightly hemolytic controls (Buna-N, 3.177%), indicating minimal erythrocyte membrane disruption. Overall, PLLA-*co*-CL/PDLA blends—particularly 3D-printed films at a 90:10 ratio—provide hydrophilic, cyto-compatible surfaces that support endothelial viability and balanced vasoactive signaling. These findings highlight their potential as biodegradable cardiovascular implant coverings.

## Graphical abstract

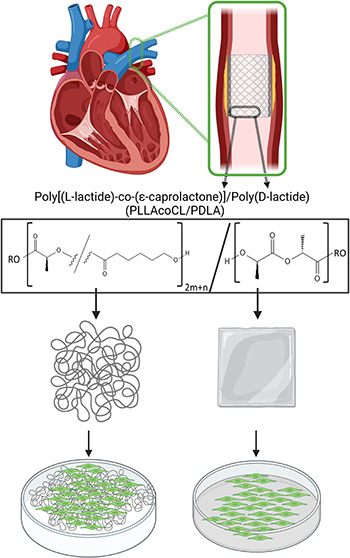

## Introduction

Cardiovascular diseases (CVDs), including heart disease and stroke, remain the leading cause of death globally, accounting for 26.8% of all deaths in 2021.^1^ Many cardiac conditions require implantation of mechanical devices such as stents, occluders, or valves, which typically combine metallic frames–commonly made from a Nitinol wire–with a flexible polymeric covering, designed to interface blood flow, providing a cyto-compatible barrier that prevents direct contact between the blood and device’s structural components, while promoting tissue integration and emboli filtration, to restore normal function and prevent life-threatening complications.^2^

Among these, the left atrial appendage occluder (LAAO) reduces stroke risk in atrial fibrillation patients and is commonly covered with expanded poly­(tetrafluoroethylene) (ePTFE) and poly­(ethylene terephthalate) (PET).^3^ While durable, these conventional polymers are nondegradable, chemically inert, and mechanically mismatched to cardiac tissue, which can trigger chronic inflammation, foreign body reactions, thrombosis, and poor endothelialization.^2,4,5^ Ideally, coverings should be biodegradable, elastomeric, and mechanically compliant (Young’s modulus ∼ 1 MPa) to support vascular cell integration and withstand dynamic cardiac stresses.^6^

Biodegradable polyesters such as polyglycolide (PGA), polylactide (PLA), poly­(ϵ-caprolactone) (PCL), and polyhydroxybutyrate (PHB) have shown promise, with PLA being widely used due to its degradability and cyto-compatibility.^7–10^ However, PLA’s brittleness and crystallinity limit its long-term performance.^11^

Recent advances with poly­[(l-lactide)-*co*-(ε-caprolactone)] (P­(LLA-*co*-CL)) highlight its toughness and cyto-compatibility for cardiovascular applications.^10,11^ Blending (P­(LLA-*co*-CL)) copolymers, with controlled L-LA sequence length, with poly­(d-lactide) (PDLA) enables stereocomplex (SC) formation for improved elasticity,^12,13^ controlled degradation,^14^ and mechanical strength–key features for next-generation cardiovascular coverings. To further justify this material’s strategy, the combination of P­(LLA-*co*-CL) and PDLA provides a synergistic architecture that cannot be achieved with either polymer alone. The flexible, low-crystallinity P­(LLA-*co*-CL) matrix contributes compliant behavior due to its glass transition temperature clearly below body temperature and high molar mass, while the short PDLA chains selectively form SC crystallites with PLLA-rich sequences. This results in a relatively small overall crystallinity that allows elastomeric behavior suitable for dynamic cardiac environments. The SC crystallites act as stable physical cross-links with high resistance against mechanical deformation and high thermal stability, reinforcing the polymer network without compromising compliance. At the same time, the system based on physical net points enables processing by 3D printing or electrospinning, which would not be the case for a system based on covalent net points. The semicrystalline phase structure furthermore modulates hydrolytic degradation: the amorphous copolymer regions degrade gradually, whereas SC domains hydrolyze more slowly, enabling a controlled and less acidic degradation profile. Such moderate degradation and improved surface stability are known to support endothelialization, reduce inflammatory responses, and enhance hemocompatibility. Therefore, the P­(LLA-*co*-CL)/PDLA system offers a tunable balance of elasticity, mechanical reinforcement, and biological performance that provides clear advantages over single-polymer systems for cardiovascular implant coverings.

These blends have, however, yet to be fully evaluated in terms of biocompatibility and functionality.^12–14^

Although other lactide-caprolactone copolymers have been extensively studied, phase structure, thermal transitions, and macroscopic behavior of polymers change quite significantly with polymer microstructure, blending, and processing; and these relationships require further understanding. The discussed material system with tailored sequence and stereocomplexation with short PDLA chains has been recently introduced by our group^12^ and has demonstrated promising results. For successful clinical applications, it needs further investigations to understand the influence of tailored material features on biological systems. In this work, we investigate these distinctive structural and functional features in the context of biomedical applications, highlighting the unique opportunities they present for cardiovascular device design.

Here, we investigated the processing of fiber-based polymer coverings based on P­(LLA-*co*-CL)/PDLA blends for cardiovascular implants by two different technologies: electrospinning and extrusion-based 3D printing. Unlike conventional films or woven textiles, electrospun submicron fiber meshes provided high surface area, porosity, and extracellular matrix (ECM)-like architecture that promotes endothelial cell (EC) adhesion, tissue integration, and antithrombotic properties. To tune the balance between elasticity and SC crystallinity/mechanical reinforcement, we evaluated the properties of various blends at 95:5, 90:10, and 85:15 ratios. The previous studies showed that low-to-moderate PDLA content can present an optimized SC while maintaining elastomeric compliance suitable for cardiovascular environments.^15–17^ Therefore, based on this know-how, we processed these blends into electrospun meshes and 3D printed films to directly compare how processing-driven morphology impacts the mechanical and biological performance of the material.

Although *ex vivo* and *in vivo* studies are ultimately required to fully evaluate cardiovascular performance, *in vitro* assays remain a critical step in the development of biomaterials. In this work, we performed a comprehensive assessment of EC behavior, going beyond routine cytotoxicity tests to include viability, metabolic activity, cytoskeletal organization, and bioactive signaling (Prostacyclin (PGI_2_), Thromboxane B2 (TXB_2_), and Nitric Oxide (NO) release). In addition, a direct-contact hemolysis assay in accordance with the principles of ISO 10993–4 was conducted as a preliminary *in vitro* red blood cell lysis test, relevant to hemocompatibility assessment. These multiple complementary assays provide robust insight into how PLLA-*co*-CL/PDLA blend composition and scaffold fabrication influence HUVECs’ behavior, establishing a strong foundation for subsequent advanced evaluations.

## Materials and Methods

### Synthesis of Poly­[(l-lactide)-*co*-(ε-caprolactone)] (PLLA-*co*-CL)

PLLA-*co*-CL with a *M*_w_ of 203 × 10^3^ g/mol (i.e., PLLA-*co*-CL203) was synthesized as reported before.^12^ Briefly, l,l-Dilactide (Purasorb L) (99.5%, Corbion, Amsterdam, Netherlands) (30.0 g, 208.2 mmol) and ε-caprolactone (99%, Th. Geyer Hamburg GmbH & Co. KG, Hamburg, Germany) (approximately 20 g, 175.2 mmol) were mixed in a 500 mL three-neck flask under argon. The flask was heated to 140 °C with constant stirring, using a magnetic stirrer. A colorless melt was obtained after 5–10 min, then Tin­(II) 2-ethylhexanoate (Sn­(Oct)_2_) (95%, ThermoFisher GmbH, Kandel, Germany) (62.1 mg, 0.15 mmol) was added dropwise through the rubber septum. The mixture was stirred for 53 h at 140 °C. After cooling to Room Temperature (RT), 400 mL of chloroform (99%, Carl Roth GmbH & Co. KG, Karlsruhe, Germany) was added, and the mixture was stirred overnight. An additional 400 mL of chloroform was added to completely dissolve the mixture, and the solution was mixed for 15 min. Afterward, the solution was precipitated in 2 L of cold methanol (at 4 °C) (99%, Carl Roth, Karlsruhe, Germany), and was left to rest for 4 h in the fridge. The methanol was then discarded, and the residue was dried in a vacuum oven at 60 °C for at least 1 day. The yield was up to 96%.

### Synthesis of Poly­(d-lactide) (PDLA)

PDLA with a *M*_w_ of 20 × 10^3^ g/mol (i.e., PDLA20) was synthesized as reported before.^12^ Briefly, d,d-dilactide (Purasorb D) (99.5%, Corbion, Amsterdam, Netherlands) (100.0 g, 690 mmol) and 1-hexanol (99%, Fisher Scientific GmbH, Hampton, New Hampshire, USA) (0.63 g, 6.2 mmol) were mixed in a dry 500 mL three-neck flask under argon. The mixture was stirred at 135 °C, then Sn­(Oct)_2_ (95%, Thermo Fisher (Kandel) GmbH, Kandel, Germany) (0.112 g, 0.28 mmol), measured with a pipet, was added dropwise through the rubber septum. The mixture was stirred for 1 h at 135 °C. After cooling at RT, 400 mL of chloroform (99%, Carl Roth GmbH & Co. KG, Karlsruhe, Germany) was added until complete dissolution of the product. The obtained solution was precipitated in 400 mL of 4 °C methanol (99%, Carl Roth, Karlsruhe, Germany) and was left to rest for 4 h in the fridge. The methanol was then discarded, and the supernatant was dried in a vacuum oven at 60 °C for at least 1 day. The yield was up to 95% of polymer.

### Physiochemical Characterization of the Synthesized Polymers

#### Functional Groups Using Nuclear Magnetic Resonance (NMR)

^1^H and ^13^C NMR Spectra were recorded at 298 K on a 700 MHz Avance Neo spectrometer equipped with a 5 mm TCI Prodigy cryoprobe (Bruker, Ettlingen, Germany) using deuterated chloroform (CDCl_3_) as a solvent. Chemical shifts (δ) were reported relative to CDCl_3_ at δ 7.26 ppm for ^1^H NMR spectra and δ 77.0 ppm for ^13^C NMR spectra.

To determine the number-average sequence length of lactide and caprolactone blocks, as well as the randomness of the copolymer, the relative dyad content together with the molar ratio of lactide and caprolactone was calculated using the following equations

1 $$l_{\mathrm{LA}}=\frac{2\left(\mathrm{LA}\right)}{\left(\mathrm{LA}-\mathrm{CL}\right)}$$

2 $$l_{\mathrm{CL}}=\frac{2\left(\mathrm{CL}\right)}{\left(\mathrm{LA}-\mathrm{CL}\right)}$$

3 $${\left(l_{\mathrm{LA}}\right)}_{\mathrm{random}}=\frac{1}{\left(\mathrm{CL}\right)}$$

4 $${\left(l_{\mathrm{CL}}\right)}_{\mathrm{random}}=\frac{1}{\left(\mathrm{LA}\right)}$$

5 $$R=\frac{\left(\mathrm{LA}-\mathrm{CL}\right)}{2\left(\mathrm{LA}\right)\left(\mathrm{CL}\right)}$$

­(LA) and (CL) represent the molar fractions of the respective monomers in the copolymer, and (LA – CL) denotes the relative molar fraction of this dyad. The number-average block lengths of the comonomers are represented by *l*_LA_ and *l*_CL_, while (*l*_LA_) random and (*l*_CL_) random refer to the Bernoulli average block lengths. The randomness parameter *R* is defined as the ratio of the Bernoulli block length to the observed block length. For a completely random copolymer, *R* = 1; for a block copolymer, *R* approaches 0.

#### Functional Groups Using Fourier Transform Infrared (FTIR) Spectroscopy

The chemical structure of the synthesized polymers was analyzed using a Nicolet 6700 FTIR spectrometer (Thermo Fisher Scientific, Dreieich, Germany). FTIR measurements were performed in transmittance mode, scanning each sample 50 times over the spectral range of 600–4000 cm^–1^ with a data resolution of 4 cm^–1^. The spectra were used to assess the presence of characteristic functional groups.

#### Molecular Weight Using Gel Permeation Chromatography (GPC)

The molecular weight characteristics of the synthesized polymers, including number-average molar mass (*M*_n_), weight-average molar mass (*M*_w_), and dispersity (*Đ*), were determined using GPC. Measurements were carried out on a 1260 Infinity II Security PSS GPC System (PSS, Polymer Standards Service, Mainz, Germany) equipped with an isocratic pump, degasser, autosampler, column oven, UV detector, and Refractive Index (RI) detector. Additional detection was performed using a BI-MwA multiangle light scattering detector (Brookhaven Instruments, Holtsville, New York) and a Security DVD 1260 differential viscometer (PSS, Polymer Standards Service GmbH, Mainz, Germany). The separation was achieved using a column setup consisting of a very stable styrene divinylbenzene (VS SDV) Lux, 10 μm, 50 mm × 8 mm inner diameter precolumn and two SDV Lux 10 μm, 300 mm × 8.0 mm ID analytical, linear extended lifetime (XL) columns (PSS, Polymer Standards Service GmbH, Mainz, Germany). Trichloromethane (TCM) was used as the solvent for polymer dissolution and elution. Data analysis was performed using WinGPC UniChrom software (Build 9050, Polymer Standards Service GmbH, Mainz, Germany), and molecular weight calibration was based on polystyrene standards with *M*_n_ values ranging from 1,820 to 975 000 g/mol.

#### Thermal Behavior Using Differential Scanning Calorimetry (DSC)

The thermal behavior of the synthesized polymers was investigated using a Netzsch DSC 204 (Selb, Germany). DSC measurements were performed under nitrogen atmosphere, by loading 5–7 mg of the sample into a pierced lid aluminum crucible. The polymers were scanned in three steps: [1] heating from 25 to 250 °C, [2] cooling to −100 °C, and [3] heating to 250 °C again with a temperature rate of 10 K/min. The melting (*T*_m_) temperature was obtained from the first heating run to evaluate the thermal properties of the samples and to identify how the crystalline microstructure changes during the degradation process.

#### Thermal Degradation Profile Using Thermogravimetric Analysis (TGA)

The thermal decomposition behavior of the synthesized polymers was analyzed using thermogravimetric analysis (TGA) on a Netzsch TGA 209 Iris (Selb, Germany). Approximately 5–7 mg of each sample was placed in a pierced-lid aluminum crucible and heated from 25 to 550 °C at a rate of 10 K/min under an air atmosphere. The resulting TGA data were used to generate derivative thermogravimetric (dTG) curves, with the decomposition temperature determined from the peak of the dTG curve, indicating the point of maximum degradation.

#### Preparation of Films for SC Formation Analysis via Solvent Casting

A total of 600–700 mg of PLLA-*co*-CL203 and PDLA20 in a 95:5 blend ratio was dissolved in 12 mL of chloroform (CHCl_3_) at 58 °C using a thermomixer set to 700 rpm for 2 h. The resulting solution was cast in a 6 cm diameter glass Petri dish to cover the surface and reach a film thickness of 0.1–0.2 mm. The cast film was kept to cool down to RT overnight. Subsequently, it was tightly covered with aluminum foil and placed under a fume hood to allow solvent evaporation for a minimum of 24 h. The film was dried further in a vacuum oven at 60 °C overnight using a membrane pump. After cooling to RT, the film was carefully removed from the dish using tweezers. These solvent-cast films were subsequently utilized for further evaluation of SC formation between PLLA-*co*-CL and PDLA.

#### Analytical Confirmation of SC Formation

The crystalline structure and confirmation of SC formation in the solvent-cast films of the individual synthesized polymers (PLLA-*co*-CL and PDLA), as well as their blends (95:5, 90:10, and 85:15), were investigated using Wide-Angle X-ray Scattering (WAXS). Measurements were performed at RT using a D8 Discover spectrometer equipped with a 2D detector (Bruker AXS, Karlsruhe, Germany) operated with Cu Kα radiation (λ = 0.154 nm) at a voltage of 40 kV and a current of 40 mA. The peak positions corresponding to SC PLA and homopolymer PLA (PLLA or PDLA) were determined with an angular resolution of Δθ = 0.1°, accounting for minor deviations due to sample thickness and placement within the holder (for flat samples).

As described above, the same parameters were used for DSC to confirm SC formation.

### Polymer Processing

#### Film Production via 3D-Extrusion Printing

A 3D-Bioplotter Developer Series (EnvisionTEC, Gladbeck, Germany) was used to print PLLA-*co*-CL203/PDLA20 (95:5, 90:10, and 85:15 blends) solutions with a total 20 wt % concentration in chloroform. STL files of 30 mm × 30 mm films were designed using Autodesk Inventor (Autodesk, California, USA), and slicing was conducted using EnvisionTEC’s Perfactory Suite software (EnvisionTEC, Gladbeck, Germany). The slicing parameters were adjusted to match 80% of the nozzle tip diameter, ensuring optimized line spacing and print fidelity based on the specific needle used. Printing was performed using conical plastic nozzle tips with diameters of 0.2, 0.25, and 0.4 mm (Nordson EFD, East Providence, USA; Precision Tip Series 7018436). Prior to finalizing the optimal conditions, a range of parameters was evaluated to achieve high-resolution structures, including solution concentration (5, 10, 15, 20, and 25 wt %), pressure (1, 1.2, 1.4, 1.6, 1.8, and 2.0 bar), printing speed (5, 10, 15, 20, 25, and 30 mm/s), printing temperature (20, 25, and 30 °C), and nozzle-tip diameter (0.2, 0.25, and 0.4 mm).

The films (30 mm × 30 mm) were fabricated using optimized parameters: 1.8 bar, 5 mm/s, 25 °C printing temperature, and 25 °C platform temperature. The printed films were dried overnight at RT and stored at 5 °C until use.

#### Fiber Mesh Production Using Electrospinning

Electrospun meshes were fabricated using a custom-made electrospinning setup from Linari Engineering (Pisa, Italy), consisting of a high-voltage power supply, a rotating easy drum entry-level (EL-D) system (diameter: 10 cm), and a programmable syringe pump (Model BSP-99M, Linari Engineering). The polymer solutions were prepared by dissolving PLLA-*co*-CL203/PDLA20 blends (95:5, 90:10, and 85:15 w/w) in chloroform at a concentration of 8 wt %. Prior to electrospinning, the solutions were filtered through a 1 μm PTFE membrane filter and loaded into a 20 mL syringe fitted with 19G stainless steel needles (catalog no. 72310–200; inner diameter: 0.69 mm; VWR International GmbH, Dresden, Germany). Electrospinning was conducted inside a transparent acrylic chamber equipped with a variable airflow system to regulate environmental conditions. Relative humidity was maintained between 13–15% and monitored using a digital thermos-/hygrometer (Distrelec Deutschland GmbH, Bremen, Germany) at a constant ambient temperature of 25 °C (RT). The needle tip-to-collector distance was fixed at 30 cm, with a solution flow rate of 2.12 mL/h. A voltage of 25–27 kV was applied, and the drum collector was rotated at a controlled speed (15 rpm) to ensure uniform fiber deposition.

The following range of parameters was tested prior to determining the optimal parameters: solution concentration (5, 8, and 10 wt %), voltage (starting with 12 kV reaching up to 25 kV), the distance between nozzle tip and collector (15, 20, 25, and 30 cm), humidity (12, 15, and 20%), flow rate (2.12 and 3.14 mL/h), and drum collector with varied rotating speed (5, 10, and 15 rpm).

### Characterization of Films and Meshes

#### Rheological Measurements

The rheological properties of PLLA-*co*-CL/PDLA solutions (95:5, 90:10, and 85:15 w/w) were investigated to assess their dynamic viscosity and shear-thinning behavior, relevant to 3D printing applications. Measurements were performed using the HAAKE MARS rheometer (Thermo Fisher Scientific, Massachusetts, USA) equipped with a Titanium cone/plate system (35 mm diameter, 2° cone angle, and truncation gap of 54 μm). The system was equilibrated at 25 °C for 10 min prior to the measurement, and 1 mL of each solution was carefully applied to the plate. Viscosity was measured under steady shear flow conditions over a range of shear rates from 0.01 to 1000 s^–1^. Each run lasted 5 min, during which 100 data points were recorded (using the HAAKE RheoWin 4 Software). A series of PLLA-*co*-CL/PDLA solutions (at 95:5, 90:10, and 85:15 blends) with concentrations of 15, 20, 25, 30, and 35 wt % in chloroform were analyzed to evaluate their shear-thinning profiles and determine their suitability for extrusion-based fabrication methods.

#### Morphology and Fiber Diameter Using Scanning Electron Microscopy (SEM)

Morphology of electrospun meshes was characterized using the Benchtop-SEM FEI Phenom G2pro (Thermo Fisher Scientific Inc., Hennigsdorf, Germany). Samples were fully dried in a vacuum and coated with 5 nm gold to ensure electrical conductivity using a sputter coater Q150R ES plus (Quorum Technologies Ltd., Laughton, UK). Fiber diameter and diameter distribution were analyzed from SEM images using ImageJ software. For each sample, at least three independent electrospun meshes (*n* = 3) were examined. From each replicate, three nonoverlapping SEM images were taken at 600, 2000, and 8000× magnification, and at least 20 individual fibers per sample were measured to determine the average diameter and distribution characteristics.

#### Mechanical Characterization of Films and Meshes

Uniaxial tensile testing of the electrospun meshes and 3D printed films was carried out using a Z1.0 tensile test apparatus (ZwickRoell GmbH & Co. KG, Ulm, Germany) with a 20 N load cell.

Samples were prepared following BS ISO 13781:2017.^7^ Each test specimen was used for one mechanical test only. Uniaxial tensile tests were carried out (*n* = 6) at RT. Samples were punched into a dog-bone shape with the following dimensions: 30 mm × 5 mm × 0.5 mm. The thickness of each sample was calculated as an average of three different measurements.

Stress–strain curves were obtained (with a strain rate of 200 mm/min), and displacement (mm) and force (N) were recorded by the software, testXpert3 (from ZwickRoell GmbH & Co., KG, Ulm, Germany).

Engineering stress (MPa) and strain (%) were calculated using eqs 6 and 7, respectively. Young’s modulus [E] (MPa) was calculated from the slope of the linear region of stress–strain curves.

6 $$ultimate\;stress\;\left[\sigma \right]\;\left(MPa\right)=\frac{ultimate\;force\;\left(N\right)}{cross‐\mathrm{sect}ional\;area\;\left(mm^{2}\right)}$$

7 $$\mathrm{ultimate}\;\mathrm{strain}\;\left[\varepsilon \right]\;\left(\%\right)=\frac{\Delta L}{L_{0}}$$

*L*_0_: initial length of the sample, and Δ*L* is the difference between the initial and final length of the sample.

### Preparation of Films and Meshes for Biological *In Vitro* Studies

#### Sterilization of Films and Meshes

All samples were sterilized in an automated ethylene oxide (EtO) sterilization system (SteriVit 100, DMB Apparatebau, Wörrstadt, Germany) under controlled conditions of 45 ± 3 °C, 68% relative humidity, and 1.7 bar pressure, for a duration of 180 min. Prior to sterilization, the samples were sealed in sterile, EtO-compatible breathable sterilization pouches (SteriClin Sterilization Bags, Carl Roth GmbH, Karlsruhe, Germany), which allow for effective EtO gas penetration and maintain sterility postprocess. Each pouch was clearly labeled with batch identification to ensure full traceability throughout sterilization and handling.

#### Wettability Using Contact Angle Measurements

Surface wettability of the electrospun meshes and 3D printed films was evaluated using static contact angle measurements via the sessile drop method. A DSA100 contact angle goniometer (KRÜSS GmbH, Hamburg, Germany) was used to assess both advancing and receding contact angles. Water droplets were dispensed onto the sample surfaces using a stainless-steel needle attached to a microliter syringe. The droplet size gradually increased and decreased from an initial diameter of approximately 3 mm to measure dynamic contact angles. All measurements were performed under ambient conditions. To ensure statistical reliability, a minimum of ten measurements were obtained from individual droplets positioned at no fewer than three distinct locations on each sample type. The results were averaged, and standard deviations were calculated to assess variability across the surfaces.

### Biological *In Vitro* Studies

#### Cell Cultivation Procedure

Human umbilical vein endothelial cells (HUVECs; Lonza, Cologne, Germany) at passage 5 were cultured in Endothelial Cell Growth Medium-2 (EGM-2 BulletKit, CC-3162 Lonza, Cologne, Germany), which includes basal medium supplemented with 2% (v/v) fetal bovine serum, vascular endothelial growth factor (VEGF), basic fibroblast growth factor (bFGF), human epithelial growth factor (hEGF), insulin-like growth factor-1 (IGF-1), hydrocortisone, heparin, ascorbic acid, gentamycin, and amphotericin B. Cells were maintained in a humidified incubator at 37 °C with 5% CO_2_ and cultured until reaching 70–80% confluency. Concurrently, HUVECs were split using Trypsin/EDTA (0.25% Trypsin +0.53 mM EDTA, Cat.No. P10–036100, PAN-Biotech GmbH, Aidenbach, Germany), counted, and seeded at a density of 5,000 cells/well in a 96-well plate (Greiner Bio-One, Frickenhausen, Germany) and incubated overnight (37 °C, humidified atmosphere at 5% CO_2_).

#### Indirect Cytotoxicity Assessment

To evaluate the potential cytotoxic effects of the samples, indirect cytotoxicity assays (based on the samples’ extracts in the supernatant), such as lactate dehydrogenase (LDH) release, metabolic activity (MTS), and cell viability, were conducted using HUVECs.

Films and Meshes (6 × 6 cm^2^) were immersed in 15 mL tube in EGM-2, as described above, and incubated for 72 h at 37 °C. For each tube containing material, 15 mL of EGM-2 was added, and the tubes were wrapped in aluminum foil and rotated on an orbital shaker (15,000 U/min) at 37 °C for 72 h. In parallel, a tube with EGM-2 only (without material) was prepared to serve as the negative control.

After 72 h of incubation, the conditioned medium (extract) was collected and stored overnight at 4 °C. The next day, the medium from the HUVECs culture was removed and replaced with either fresh EGM-2 (negative control), extract medium (undiluted, 1:0 and diluted, 1:10), or medium supplemented with Cell Lysis Solution after 48 h (containing 1 mM CuCl_2_ in standard medium) (serving as positive control). The HUVECs were cultured with the sample-derived medium for 48 h. Subsequently, assays were performed to evaluate cell behavior as follows:

### Cell Membrane Integrity via Measuring Lactate Dehydrogenase (LDH) Release

Cell membrane integrity was measured using a Lactate Dehydrogenase (LDH) Cytotoxicity Kit II (BioVision, California, USA) following the manufacturer’s instructions. Briefly, 50 μL of cell culture supernatant from each well was mixed with 50 μL of LDH reaction mixture and incubated for 10 min at RT in the dark. The absorbance was measured at 450 nm (with a reference wavelength of 650 nm) using a microplate reader (Tecan Infinite M200 Pro, Crailsheim, Germany). Results represent the mean value of three independent experiments; each performed with three biological replicates and four technical replicates (*n* = 36).

### Metabolic Activity

The metabolic activity of HUVECs was evaluated using an MTS cell Titer 96 Aqueous Nonradioactive Cell Proliferation Assay (MTS assay, Promega, Mannheim, Germany), according to the manufacturer’s instructions. Briefly, 20 μL of MTS mixture was added to 100 μL cell culture media in a 96-well plate and was incubated for 3 h at 37 °C (humidified atmosphere at 5% CO_2_). The absorbance of each well was then measured at a wavelength of 492 nm using a microplate reader (Tecan Infinite M200 Pro, Crailsheim, Germany). The mean of absorbance of six replicates and the standard deviation were calculated.

### Cell Viability via FDA/PI Staining

Cell viability evaluation was performed using fluorescein diacetate (FDA; Sigma-Aldrich, Taufkirchen, Germany, stock solution 25 mg/mL), propidium iodide (PI; Invitrogen, California, USA; stock solution 1 mg/mL in water), and Hoechst 33342 (Invitrogen, California, USA; stock solution 10 mg/mL in water). FDA was added to the cell culture medium at a 1:200 dilution, and PI and Hoechst were each added to the cell culture medium at a 1:500 dilution. All stains were simultaneously added to the culture medium of the seeded wells. Images of the cells were taken using the automated InCell Analyzer 200 (GE Healthcare, Chicago, USA), and the number of live (green) and dead (red) cells was quantified automatically using the ImageJ software. The cell viability was measured per image, and the mean of six replicates and the standard deviation were calculated.

### Direct Cytocompatibility Studies

#### Cell Cultivation Procedure

HUVECs were seeded at a density of 15,000 cells/well onto sterilized film and mesh samples in nonadherent 24-well tissue culture plates (TCP; Thermo Fisher Scientific, Roskilde, Denmark). To prevent floating of the samples during culture, the 1 cm^2^ specimens were fixed using autoclaved cell crown inserts (Scaffdex, Tampere, Finland).

Samples (replicates = 3) were fabricated from PLLA-*co*-CL/PDLA blends at ratios of 95:5, 90:10, and 85:15 and sterilized according to the procedure described above. Negative controls (cells in culture medium only) and positive controls (cells treated with 0.5% Triton (v/v) (Sigma-Aldrich, Taufkirchen, Germany) for 10 min shortly before the staining) were cultured in separate adherent 24-well TCPs (TPP Techno Plastic Products AG, Trasadingen, Switzerland). Cultured samples were maintained for different time points with medium changes every other day.

#### Cell Viability Assessment

Cell viability on electrospun meshes and 3D printed films was assessed at three time points–days 1, 3, and 7 of culture-using a dual-staining fluorescence assay. To directly label the cells on the samples, FDA (Sigma-Aldrich, Taufkirchen, Germany, stock solution 5 mg/mL), for viable cells, and PI (Invitrogen, California, USA; stock solution 1 mg/mL in water) for nonviable cells were freshly prepared in phosphate-buffered saline (PBS, pH 7.4; Sigma-Aldrich Chemie GmbH, Taufkirchen, Germany) and applied directly onto the cell-seeded samples in each well, ensuring complete surface coverage. Fluorescence images were acquired immediately after staining using an Axio Vert.A1 inverted fluorescence microscope (Zeiss, Jena, Germany) at 10× magnification.

Qualitative analysis of live (green) and dead (red) cells was performed using Fiji (ImageJ, version 1.48c), and results were expressed as the percentage of viable cells relative to the total cell count. For each condition, three independent experiments were conducted, with 12 images analyzed per experiment, taken from three different wells.

#### Metabolic Activity Using the MTS Assay

The proliferation rate and metabolic activity of the HUVECs cultured on the samples, as described above, were assessed over 7 days at defined time points (days 1, 3, and 7). At each time point, 100 μL of the MTS reagent (CellTiter 96 Aqueous One Solution Cell Proliferation Assay, Promega, Mannheim, Germany) was added directly to 500 μL of cell culture medium in each well of the 24-well plate, and incubated for 3 h at 37 °C. For all measurements, independent samples were used for each time point (i.e., destructive sampling). Metabolic activity was normalized to the number of viable, adherent cells/mm^2^. Data represent mean values from three independent experiments performed in triplicate (*n* = 9).

#### Cytoskeleton Analysis Using the Actin and Nuclei Staining

To evaluate the morphology and alignment of ECs cultured on electrospun meshes and 3D printed films, fluorescence staining of the cytoskeleton and nuclei was performed following 1, 3, 7, 10, and 14 days of incubation. Adherent cells were initially rinsed twice with PBS, fixed with 4% paraformaldehyde (PFA; Merck KGaA, Darmstadt, Germany) for 15 min at RT, and subsequently permeabilized with 0.1% Triton X-100 (Sigma-Aldrich, Taufkirchen, Germany) in PBS for 5 min at RT. For staining, 500 μL of ROTIMount FluorCare containing 4′,6-diamidino-2-phenylindole (DAPI; 0.1 mg/mL; Carl Roth GmbH, Karlsruhe, Germany) was used for nuclear labeling—targeting adenine- and thymine-rich regions of DNA- and was mixed with 250 μL of Phalloidin Dylight 488 (MAN 0001777; Invitrogen, California, USA) for selective labeling of filamentous actin (F-actin) in 10 mL of PBS. Samples were incubated in the staining solution for 30 min at RT in the dark. Afterward, samples were rinsed once with PBS and immediately imaged using a fluorescence microscope (Axio Vert, Zeiss, Jena, Germany).

#### Direct-Contact Hemolysis Assay

The hemolytic potential of the samples was evaluated using a manual hemolysis assay in accordance with the principles of ISO 10993–4 for interactions with blood. Human erythrocyte suspension was obtained from a commercial kit (Hemolysis Assay for Biomaterials, K003, Lot # 251006, Hemoscan BV, Groningen, Netherlands). The erythrocyte suspension was washed and resuspended in PBS without Ca^2+^/Mg^2+^ and used as the working suspension. A positive control inducing complete hemolysis was prepared by diluting the erythrocyte suspension 1:10 in deionized water (DI), while PBS-diluted erythrocyte suspension served as the negative control. Reference samples included nonhemolytic silicone elastomer and slightly hemolytic Buna-N (nitrile rubber, both Hemoscan BV, Groningen, Netherlands).

Samples were cut to size (0.5 cm × 1.5 cm), placed in 1.5 mL microcentrifuge tubes, and 500 μL of the erythrocyte working suspension was added to each tube. Samples were incubated for 24 h at 37 °C under gentle end-overend rotation to ensure continuous contact between the test material and erythrocytes. Following incubation, samples were centrifuged at 4000*g* for 1 min to separate intact erythrocytes from the supernatant containing released hemoglobin. Per sample, 3 × 100 μL were transferred to a 96-well plate, and absorbance was measured at 540 nm using a microplate reader (Infinite M Plex, Tecan, Männedorf, Switzerland). Hemolysis was determined by correcting all absorbance values for spontaneous hemolysis by subtracting the absorbance (Abs) of the negative control (PBS-diluted erythrocyte suspension). Corrected absorbance values of test samples were expressed as a percentage of the positive control (DI-diluted erythrocyte suspension), representing complete hemolysis.

8 $$\mathrm{hemolysis}\;\left(\%\right)=\frac{{\mathrm{Abs}}_{\mathrm{sample}}-{\mathrm{Abs}}_{\mathrm{negative}\mathrm{control}}}{\mathrm{average}\;\left({\mathrm{Abs}}_{\mathrm{positive}\mathrm{control}}-{\mathrm{Abs}}_{\mathrm{negative}\mathrm{control}}\right)}$$

#### Quantification of Vasoactive Substance Release from Cell-Seeded Samples

This section describes the quantification of endothelial-derived vasoactive substances—namely Nitric Oxide (NO), Prostacyclin (PGI_2_), and Thromboxane B2 (TXB_2_)—secreted by HUVECs on polymeric films and meshes over defined time points.

#### Sample Preparation and Supernatant Collection

HUVECs were cultured with 20,000 cells/well directly on polymeric films and meshes following the procedure detailed methodology. Culture supernatants were collected after 1, 3, and 7 days to assess the secretion of vasoactive mediators. To eliminate interference from fetal bovine serum (FBS) in downstream enzyme-linked immunosorbent assays (ELISAs), the supernatants were subjected to ultrafiltration using 10 kDa molecular weight cutoff centrifugal filter tubes (Amicon, Merck KGaA, Darmstadt, Germany), prewetted with ultrapure water (Cayman Chemicals, Hamburg, Germany). Filtration was performed at 4000*g* for 20 min at RT. The resulting FBS-free supernatants were stored at −80 °C.

To enhance detection sensitivity, filtered supernatants were lyophilized for 24 h using a freeze-dryer (α 1–2 LD Plus, Martin Christ, Osterode, Germany) and stored at −20 °C.

Prior to analysis, they were reconstituted with ultrapure water to one-third of their original volume, and this concentration factor was incorporated into all subsequent calculations.

#### Quantification of Nitric Oxide (NO) Production in HUVECs

Nitric oxide production was indirectly assessed by measuring its stable end products, nitrate and nitrite, using the Total Nitric Oxide and Nitrate/Nitrite Parameter Assay Kit (R&D Systems, Wiesbaden, Germany), according to the manufacturer’s instructions.

Briefly, in a 96-well microplate format, standards and blank controls were prepared in parallel with samples. The calibration curve was established from a serial dilution of supplied standards, covering the expected concentration range. Sample measurements were performed using 50 μL of rehydrated supernatant in technical triplicate. The assay was carried out in two phases: [1] endogenous nitrite, which was quantified directly, and [2] total nitrite, which was measured via colorimetric detection (Griess reaction) after enzymatic conversion of nitrate to nitrite using nitrate reductase. Absorbance was recorded at 540 nm (reference: 690 nm) using a microplate reader. Data processing and concentration determination were performed following the manufacturer’s protocol, which calculates concentrations from a generated standard curve.

#### Quantification of Prostacyclin and Thromboxane B2 Production in HUVECs

The levels of PGI_2_ (also known as 6-keto Prostaglandin F_1_α) and TXB_2_ were determined using two separate competitive ELISA kits—6-keto PGF1α ELISA Kit (Abbexa Ltd., Cambridge, UK) and Thromboxane B_2_ ELISA Kit (Cayman Chemical, Hamburg, Germany). Assays were conducted according to the respective manufacturer’s protocols. For each assay, full standard curves (supplied standard dilutions) and blanks were prepared in designated wells of a 96-well plate alongside the test samples (50 μL each), which were measured in triplicate.

The absorbance was recorded at 450 nm using a microplate reader. Quantitative analysis was performed using the Excel-based analysis sheet provided by the manufacturer, which included a standard curve fitting algorithm (typically 4-parameter logistic regression). Final concentrations were normalized to the number of vital adherent cells per mm^2^, based on counts from three independent experiments (*n* = 9).

#### Statistical Analysis

All results were treated using the software OriginPro 2024 (OriginLab Corporation, Northampton, MA). Graphical data representations, including charts and error bars, were used to visualize the mean values and the variability among experimental groups. All experimental data were statistically analyzed to evaluate the reproducibility and significance of the results. For each experimental condition, at least three independent samples were tested to ensure consistency and reliability. Results are expressed as mean ± standard deviation (SD) when data are normally distributed, and statistical relevance is established; otherwise, the median along with minimum and maximum values is reported.

## Results and Discussion

### Physiochemical Properties of the Synthesized PLLA-*co*-CL and PDLA

To verify that the synthesized polymers possessed the intended chemical structures, a series of structural characterization analyses were conducted. This verification was critical to ensure that any observed differences in physical properties or biological responses can be reliably attributed to differences in polymer composition or processing conditions, rather than to inconsistencies or errors in the synthesis process.

PLLA-*co*-CL was synthesized as described in Materials and Methods section, with the synthesis route shown in Figure 1a. Based on eqs 1 and 2, the copolymer contained lactide (LA) and caprolactone (CL) block lengths (*l*_LA_, *l*_CL_) of 8.64 and 3.12, respectively. PDLA was synthesized as shown in Figure 1b, and as described in Materials and Methods section. Both polymers were characterized using ^1^H NMR and ^13^C NMR (Figures 1c and S1) to confirm their molecular structures. The molar ratio of LA to CL (LA/CL) in the copolymer was determined from the integral ratios of characteristic peaks in the ^1^H NMR spectra (Figure 1c) with CH signals from LA compared to –OCH_2_ or −CO–CH_2_ signals of CL (Table 1). Additionally, the ratio of LA-LA and LA-CL dyads was calculated to determine the number-average sequence lengths of each block and Bernoulli average block lengths (eqs 1–4). The copolymer’s randomness (*R*) was calculated using eq 5, and the resulting values are summarized in Tables 1 and 2.

**Figure 1:**
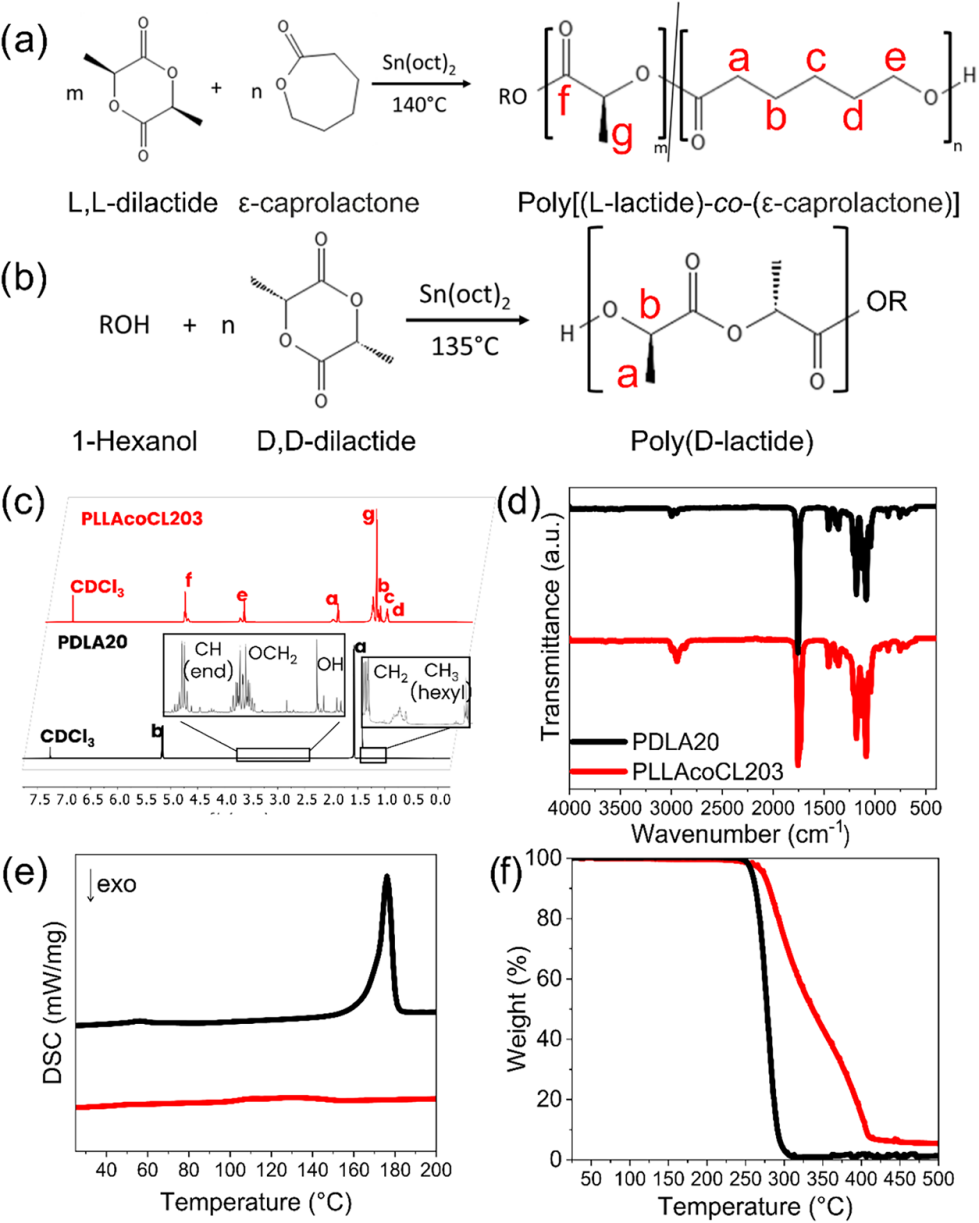
Synthesis Routes for (a) Poly­[(l-lactide)-*co*-(ε-caprolactone)] (PLLA-*co*-CL) and (b) Poly­(d-lactide) (PDLA). Physiochemical properties of the synthesized PLLA-*co*-CL and PDLA: (c) ^1^H NMR spectra, (d) FTIR transmission spectra, (e) first heating DSC curves, and (f) TGA curves of the synthesized PLLA-*co*-CL (red) and PDLA (black) with *M*_w_s of 203 and 20 kg/mol, respectively.

**Table 1:** Proton Nuclear Magnetic Resonance (^1^H NMR) Analysis of PLLA-*co*-CL Copolymer with *M*_w_ of 203,000 g/mol, Highlighting Chemical Composition and Structural Characteristics^a^

PLLA-*co*-CL203, LA/CL ratio (73.5:26.5) *l*_(LA–LA)_ = 8.64; *l*_(CL–CL)_ = 3.12; *R* = 0.434			
δ (ppm)	multiplicity	coupling constant (^3^ *J*) [Hz]	LA-conversion = 99.9% CL-conversion = 99.9% ref. to structure
5.18–5.14	q	7.3	CH LA–LA diad
5.14–5.09	m	-	CH LA–CL diad, partially overlapping with LA–LA diad
4.16–4.09	m	-	O–CH_2_ CL–LA diad
4.09–4.04	t	6.7	O–CH_2_ CL–CL diad
2.42–2.35	m	-	CO–CH_2_ LA–CL diad
2.31–2.28	t	7.6	CO–CH_2_ CL–CL diad
1.70–1.3	-	-	CH_2_ and CH_3_

a Chemical Shift (δ), Block Length (LA) (*l*_(LA–LA)_), Block Length (CL) (*l*_(CL–CL)_), Randomness (*R*).

**Table 2:** Proton Nuclear Magnetic Resonance (^1^H NMR) Analysis of PDLA Homopolymer with *M*_w_ of 20,000 g/mol, Detailing Chemical Composition and Structural Properties^a^

PDLA 20; calculated*M*_n_ = 12,500 g/mol			
δ [ppm]	multiplicity	coupling constant (^3^ *J*) [Hz]	ref. to structure
5.18–5.16	q	7.1	CH–Lactide
4.35	q	6.9	CH terminal
4.16–4.10	m	-	OCH_2_
1.58	d	7.1	CH_3_–Lactide
1.52–1.48	m	-	CH_2_ Hexyl
0.88	t	6.9	CH_3_ Hexyl

a Chemical Shift (δ).

Representative FTIR spectra of PLLA-*co*-CL and PDLA are presented in Figure 1d. The O–H stretching band at 3507 cm^–1^ confirms the presence of PLA hydroxyl groups, while shared PLA and PCL bands were observed at 2998 cm^–1^ (C–H stretch), 1754 cm^–1^ (C=O stretch), and 1184 cm^–1^ (C–O stretch). Additional PLA-specific signals appeared at 1459, 1378, and 1089 cm^–1^. Similar bands in PDLA were detected at 3515, 2998, 1757, 1184, 1457, 1378, and 1089 cm^–1^. The FTIR spectra confirmed the successful synthesis of PLLA*co*CL and PDLA, showing all characteristic absorption bands consistent with expected PLA and PCL structures. These findings closely match those reported by Neffe et al. and are consistent with previously reported data for semicrystalline PLA, collectively supporting the structural integrity of the synthesized polymers.^12,18–20^

To determine the molar mass distributions, GPC was used, and the weight-average molar mass (*M*_w_), number-average molar mass (*M*_n_), and dispersity index (*Đ*) of PLLA-*co*-CL were calculated as 203.0 kDa, 69.0 kDa, and 2.9, respectively (Figure S2a and Table 3). PDLA exhibited an *M*_n_ of 12.5 kDa, *M*_w_ of 18.0 kDa, and *Đ* below 1.7 (Figure S2a and Table 3). GPC data indicate a copolymer with a molar mass profile consistent with literature, supporting reproducibility and suitability of these materials for further biomedical evaluation, as shown in comparable works, such as Neffe et al.^12^

**Table 3:** *M*_n_, *M*_w_, and *Đ* of the Synthesized PLLA-*co*-CL and PDLA Obtained from GPC Measurements Using Trichloromethane (TCM) as the Solvent^a^

sample ID	*M*_n_ (g/mol)	*M*_w_ (g/mol)	*Đ*
PDLA20	17,800	20,540	1.15
PLLA-*co*-CL203	69,000	203,000	2.94

a Number-average molar mass (*M*_n_), Weight-average molar mass (*M*_w_), Dispersity (*Đ*).

The structural and molecular characteristics of the synthesized PLLA-*co*-CL and PDLA align well with values reported in previous studies, confirming successful synthesis with expected composition and sequence distribution.^12^

The thermal properties and composition of the polymers, including the thermal degradation profile of PLLA-*co*-CL and PDLA, were examined using DSC and TGA (Figure 1e,f), and derivative TGA graphs are represented in Figure S2b. Melting temperatures (*T*_m_) for PLLA-*co*-CL and PDLA were determined from the first heating curve of DSC (Figure 1e), to capture the thermal properties of the polymers in their original crystalline state prior to any thermal history-induced changes. The glass transition temperature (*T*_g_) for PLLA-*co*-CL was determined to be 5 °C. *T*_m_’s were observed at approximately 50–60 °C for PCL and 131–137 °C for PLA, while PDLA exhibited melting peaks above 172 °C. PDLA did not display a detectable *T*_g_, which is common for semicrystalline polymers with low to moderate crystallinity, as the heat capacity change at *T*_g_ can be subtle. Here, the crystallinity of PDLA20 was calculated to be approximately 10.7%, indicating partial crystallinity rather than a highly crystalline structure.^21^ We detected the thermal degradation of PLLA-*co*-CL in two stages, reflecting the degradation of its PLA and PCL components: PLA degraded at approximately 290 °C, accounting for 50–60% of the mass, while PCL degraded near 400 °C, contributing 40–50% of the mass (Figure 1f). PDLA degraded at a temperature similar to PLA, around 280 °C. All results are summarized in Table 4.

**Table 4:** Thermal Properties of PLLA-*co*-CL203 and PDLA20 Determined via DSC and TGA: Melting Temperature (*T*_m_), Glass Transition Temperature (*T*_g_), Crystallinity (*X*_c_), and Decomposition Temperature^a^

					decomposition temperature (°C)	mass %	decomposition temperature (°C)	mass %
sample ID		*T*_m_ (°C)	*T*_g_ (°C)	*X*_c_ (%)	PLA		PCL	
PLLA-*co*-CL203	PCL PLA	61 132	5	-	295	59.3	405	40.7
PDLA20		172	-	10.65	282	-	-	-

a Melting Temperature (*T*_m_), Glass Transition Temperature (*T*_g_), Crystallinity (*X*_c_).

Thermal analyses by DSC and TGA further validated the materials’ expected behavior, showing distinct melting and degradation profiles corresponding to the individual polymer components. The observed transitions and degradation steps indicate appropriate phase composition and crystallinity, aligning well with prior literature and confirming the reproducibility of the synthesis route.^22,23^

### Analytical Detection of SC Formation in Solvent-Cast Films

To evaluate the formation of SCs in blends of PLLA-*co*-CL and PDLA, solvent casting was performed on both individual polymers (PLLA-*co*-CL, PDLA) and their blends (95:5, 90:10, and 85:15), followed by analytical characterization using WAXS (Figure 2a) and DSC (Figure 2b).

**Figure 2:**
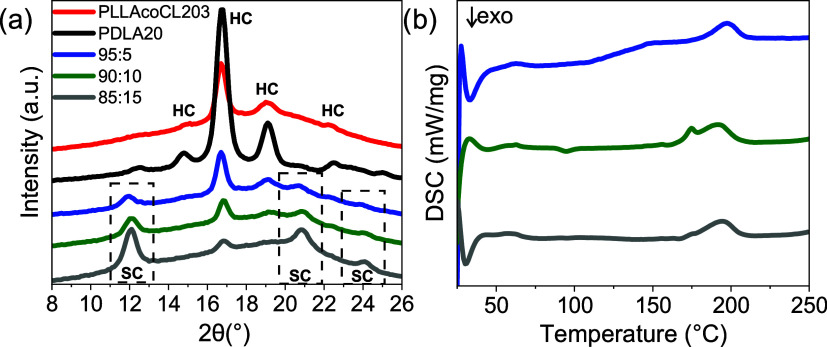
Analytical detection of stereocomplex (SC) and homocrystal (HC) formation via (a) WAXS patterns of solvent-cast films of individual polymers PLLA-*co*-CL (red) and PDLA (black), and their blends at 95:5 (blue), 90:10 (green), and 85:15 (gray) weight ratios; and (b) DSC first heating curves of the corresponding solvent-cast blends, highlighting thermal transitions associated with SC and HC crystallites.

WAXS analysis of the solvent-cast films revealed distinct diffraction peaks corresponding to PLA homocrystals (HCs) at 15°, 17°, and 19°, along with additional reflections at 21° and 22.5°, consistent with semicrystalline PLA domains. In contrast, the PLLA-*co*-CL/PDLA blends exhibited new peaks at 12°, 21°, and 24°, which are all well-established indicators of SC formation. These SC-related peaks align well with literature reports, such as those by Tsuji et al.^20^ and Brzeziński et al.,^24^ where SCs between PLLA and PDLA were confirmed by the same peak signatures.

Complementary DSC thermograms of the blends exhibited high-melting transitions around 200 °C, significantly higher than the 178 °C melting point of PLA’s HC. The elevated *T*_m_ aligns with expectations for SC crystals, reflecting their enhanced thermal stability.

Moreover, increasing PDLA content led to a proportional increase in both the absolute quantity and prevalence of SC relative to HC, mirroring trends reported in prior studies. For example, Tsjui et al. demonstrated that higher d-lactide ratios promote SC crystallization, resulting in more pronounced SC peaks and melting transitions.^20^

The combined WAXS and DSC analyses provide clear evidence for SC formation in solvent-cast PLLA-*co*-CL/PDLA blends. WAXS patterns revealed characteristic diffraction peaks for PLA HCs at 15°, 17°, and 19°, and importantly, additional peaks at 12°, 21°, and 24°, which are widely accepted as indicative of SCs.^20,24,25^

### Processing the Blend Copolymers Using 3D Printing and Electrospinning

The synthesized and prepared blend copolymers were processed using 3D printing and electrospinning. Prior to these processing techniques, the rheological behavior of the solutions was assessed, which supported optimization of the solution concentrations as well as the processing parameters.

It should be noted that both 3D printing and electrospinning inherently generate structures at different scales–electrospinning produces submicron fibrous networks, whereas 3D printing yields continuous films that, in addition to their bulk morphology, can also exhibit distinct microscale surface features such as striations or roughness arising from the layer-by-layer deposition process. Therefore, comparisons between the two formats should be interpreted as reflecting not only the influence of composition and overall fabrication scale, but also the different types of surface architectures intrinsic to each method.

### Rheological Behavior of the Blend Solutions

Prior to printing, the rheological properties of PLLA-*co*-CL/PDLA blend solutions were thoroughly characterized to assess their flow behavior under varying shear conditions (Figure 3a). Viscosity control is critical, as excessive viscosity can cause undesirable spreading of the material, while low viscosity can prevent proper material deposition.^26^ Five different concentrations (10, 15, 20, 25, and 30 wt %) at a 95:5 blend ratio were examined via frequency and amplitude sweeps, as well as shear rate-dependent viscosity measurements (additional data for 90:10 and 85:15 blends shown in Figure S3a,b).

**Figure 3:**
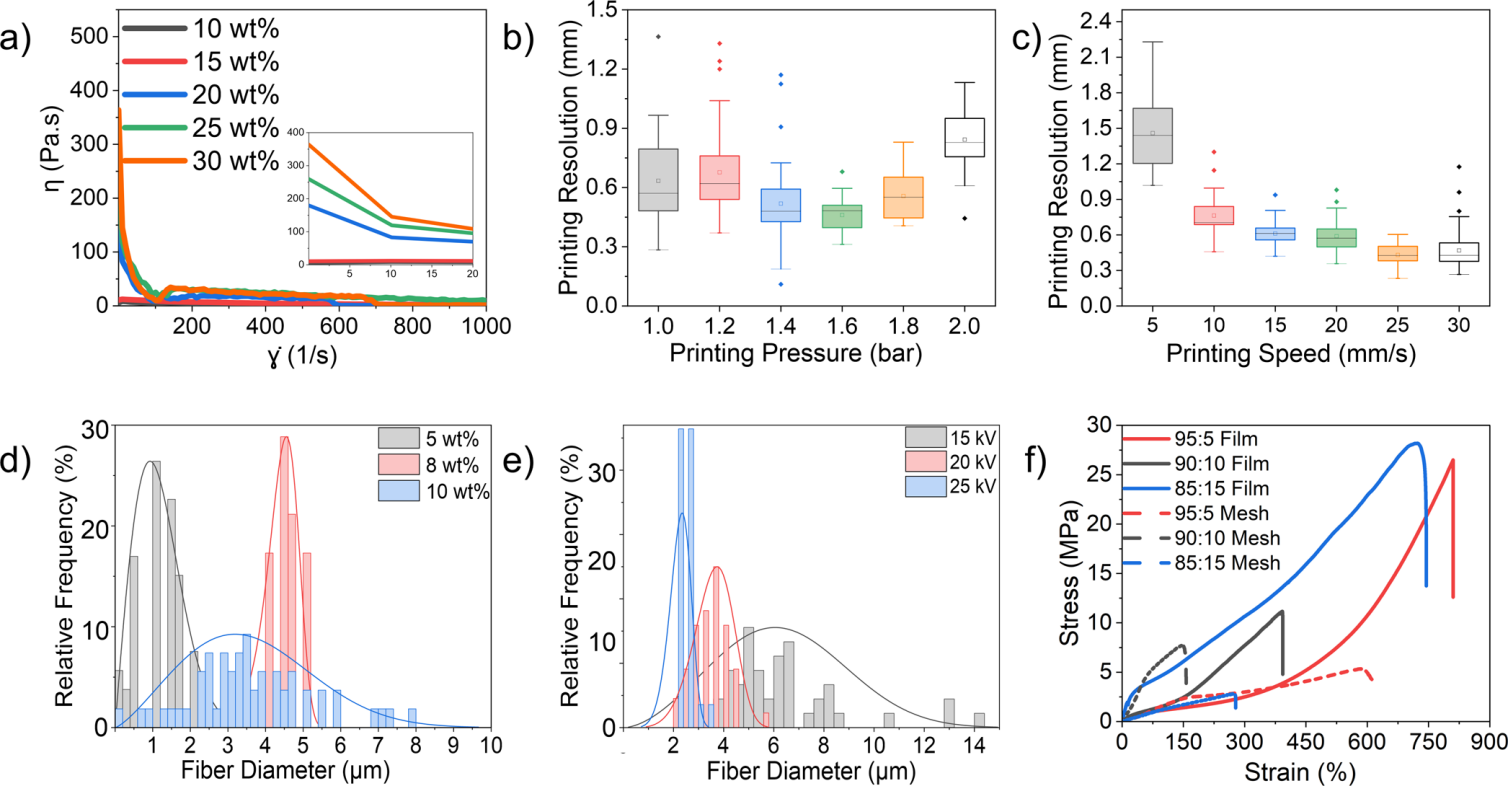
(a) Rheological properties of solutions with varying concentrations (10, 15, 20, 25, and 30 wt %) of the 95:5 PLLA-*co*-CL/PDLA blend in chloroform. The inset displays the viscosity response in the lower shear rate range (0–20 s^–1^) for improved visualization of differences at low deformation rates. Parameter optimization for 3D printing and electrospinning using the 95:5 PLLA-*co*-CL203:PDLA20 blend dissolved in chloroform: (b) effect of printing pressure (1.0, 1.2, 1.4, 1.6, 1.8, and 2.0 bar) and (c) printing speed (5, 10, 15, 20, 25, and 30 mm/s) on printed line thickness. Fiber diameter distributions as a function of (d) polymer concentration (8, 10, and 12 wt %) and (e) applied voltage (15, 20, and 25 kV) during electrospinning. (f) Static tensile mechanical properties, averaged from three samples each, for electrospun meshes and 3D-printed films.

A shear-thinning, non-Newtonian behavior, where viscosity decreases with increasing shear rate, was observed in high-concentration solutions, a desirable trait for extrusion-based 3D printing. This behavior allows the material to flow easily through the nozzle under high shear, while retaining shape postdeposition.

In Figure 3a, 30 wt % concentration (orange), nozzle clogging occurred; thus, this concentration was not selected. Solutions with lower concentrations (10 and 15 wt %, black and red) did not exhibit shear-thinning behavior, as viscosity remained constant at both low and high shear rates. A 25 wt % solution (green) exhibited high viscosity, clogging within the nozzle, compared to the 20 wt % solution (blue), which exhibited the most favorable viscosity profile, enabling consistent flow without clogging, making it the optimal concentration for 3D printing. Similar rheological trends were observed for the 90:10 and 85:15 PLLA-*co*-CL/PDLA blends across varying concentrations (Figure S3a,b). At 10 and 15 wt %, both blends exhibited low and relatively constant viscosities, similar to the 95:5 blend, indicating insufficient shear-thinning behavior for stable extrusion. At higher concentrations (25 and 30 wt %), the viscosity increases markedly, with the 30 wt % solutions again leading to nozzle clogging, comparable to observations in the 95:5 formulation. Among all tested concentrations, the 20 wt % solution consistently demonstrated the most favorable rheological profile across all blend ratios, offering balanced viscosity for stable extrusion without clogging, confirming it as the optimal concentration for 3D printing.

### Parameter Optimization for 3D Printing and Electrospinning

Beyond the novelty of the material system itself, this work also explores how different fabrication strategies–3D printing and electrospinning–shape the properties of PLLA-*co*-CL/PDLA blends for cardiovascular coverings. While both methods have individually been applied to polyester-based biomaterials, examining them side-by-side allows the influence of blend composition (stereocomplexation) to be distinguished from processing-driven morphology. From a fabrication perspective, this comparison underscores how material formulation and processing scale converge to dictate mechanical compliance and endothelial responses, thereby providing a versatile design framework for degradable cardiac coverings.

To achieve high-resolution printing, a 20 wt % solution of 95:5 PLLA-*co*-CL/PDLA blend in chloroform was selected for 3D printing. This concentration provided an ideal balance of viscosity and shear-thinning behavior, allowing for smooth extrusion through the nozzle without clogging, while maintaining sufficient structural integrity after deposition. Rheological analysis confirmed that 20 wt % consistently offered the most stable and reproducible flow properties suitable for extrusion-based printing. Notably, polyester elastomers have been successfully fabricated using techniques such as electrospinning and 3D printing, further validating our dual-fabrication strategy.^27^

For electrospinning, an 8 wt % solution of the same blend in chloroform was identified as optimal for generating uniform, bead-free fibers with controlled diameters. This concentration ensured adequate chain entanglement and viscosity to support continuous fiber formation, while enabling efficient elongation under the applied electric field. Parameters for other blend ratios (i.e., 90:10 and 85:15) were subsequently adjusted based on this optimized baseline.

For 3D printing, optimization of extrusion pressure and printing speed (Figure 3b,c) was conducted to ensure precise control over line resolution and thickness, critical for achieving uniform and high-quality 3D printed structures. Extrusion pressure (Figure 3b) affects the amount of material deposited, while printing speed (Figures 3c and S4b) influences the interaction between extrusion rate and material placement, both of which are crucial for maintaining consistent line geometry.^28^

The rheological behavior of the polymer solution, characterized by shear-thinning properties, plays a key role in this process. Under shear during extrusion, the viscosity decreases, allowing the material to flow more easily through the nozzle. However, at low extrusion pressures, the viscosity may remain relatively high, leading to unstable flow and discontinuous lines. This was observed at 1.6 bar, where, despite achieving a narrow line width (0.4 mm), the printed lines showed inconsistency and breaks (Figure S4a), likely caused by insufficient material flow due to the viscosity not dropping enough to support continuous extrusion. Increasing pressure to 1.8 bar enhanced flow stability by overcoming this viscous resistance, resulting in more uniform and continuous lines with a slight increase in thickness (0.45 mm). At 2.0 bar, the flow became excessive, causing line spreading and increased thickness (0.7 mm), reflecting a drop in printing resolution due to overdeposition.

Printing speed optimization (Figures 3c and S4b) was also critical to match the extrusion flow with nozzle movement, avoiding material accumulation or insufficient deposition. At lower speeds (5–10 mm/s), long nozzle dwell time allowed more material to accumulate, resulting in wider lines (up to 1.5 mm). Conversely, at high speeds (20–30 mm/s), the rapid movement reduced line thickness (down to 0.45 mm) but increased the risk of under-deposition and irregularities due to insufficient material supply relative to nozzle displacement. The shear-thinning rheological behavior again facilitated smoother flow at higher shear rates induced by faster extrusion and nozzle movement, enabling finer lines. A speed of 25–30 mm/s provided the narrowest and most consistent lines, making it preferable for high-resolution printing.

The effect of solution concentration (Figure 3d) and applied voltage (Figure 3e) on fiber diameter was evaluated through fiber diameter distribution analysis, plotted as relative frequency (%) and fitted with Gaussian curves to assess uniformity and central tendency. Supporting SEM images are provided in Figure S4c,d.

Solution concentration directly influences the rheological properties of the electrospinning solution, particularly polymer chain entanglement and viscosity, which are critical for stable jet formation. At 5 wt %, the solution exhibited insufficient chain entanglement and low viscosity, leading to frequent bead formation and irregular fibers with an average diameter of around 1 μm (Figures 3d and S4c). Increasing concentration to 10 wt % improved polymer chain interactions and viscosity to an optimal level that supported stable jet elongation. This resulted in thicker fibers (∼4.5 μm) with a narrow diameter distribution, indicating enhanced uniformity and process stability. At 12 wt %, viscosity increased further, causing disruption to stable jet flow and increased variability in fiber diameter (broader distribution centered at ∼3.5 μm, Figure 3d), likely due to jet instability or partial clogging. Therefore, 8 wt % was identified as the optimal concentration, balancing sufficient viscosity for continuous fiber formation with minimal structural defects, thereby maximizing reproducibility and morphological consistency.

Applied voltage (Figures 3e and S4d) influences the electrostatic stretching force on the polymer jet, impacting fiber thinning and jet stability. At 15 kV, the fiber diameter distribution was broad and centered around 7 μm, indicating inconsistent jet formation and unstable electrospinning conditions. At 20 kV, the distribution narrowed, with the average diameter centered at approximately 4 μm, suggesting improved control over fiber morphology. At 25 kV, the distribution became markedly narrower, with a peak centered around 2 μm, reflecting high process stability and uniform fiber formation. The tight Gaussian distribution at 25 kV indicates minimal variation in fiber diameter and reduced structural defects (Figure S4d), making 25 kV the optimal voltage.

The systematic optimization of processing parameters for 3D printing and electrospinning significantly improved the fidelity and reproducibility of polymeric constructs based on PLLA-*co*-CL/PDLA blends. For 3D printing, an extrusion pressure of 1.8 bar and a printing speed of 15 mm/s provided the best balance between printing resolution, material flow, and structural consistency, as supported by previous studies demonstrating the critical influence of pressure-speed coordination on filament integrity and dimensional accuracy.^29^ Similarly, for electrospinning, an 8 wt % solution concentration and 25 kV applied voltage yielded the most uniform, defect-free fibers with narrow diameter distributions, confirming trends reported by other researchers where optimal electrospinning parameters are shown to minimize bead formation and enhance fiber homogeneity.^30^ With all these parameters optimized, representative images of the final constructs for both 3D printed film and electrospun mesh are shown in Figure S5a,b, respectively.

Together, these optimized conditions enabled the reliable fabrication of tailored structures with well-defined morphologies, laying a robust foundation for future applications in biomedical scaffolds and advanced functional materials.

It is important to note that electrospinning and extrusion-based 3D printing inherently generate constructs with distinct structural characteristics: electrospinning produces nanoscale fibrous meshes, whereas 3D printing yields continuous films that also present microscale surface features, such as striations and local roughness, resulting from the layer-by-layer deposition process. While these morphologies are therefore not directly comparable, analyzing both under identical blend compositions enables the decoupling of material-driven effects (composition and SC formation) from processing-driven morphology. This dual-fabrication approach was therefore necessary to evaluate how fabrication scale impacts both the mechanical performance and EC responses, providing a more comprehensive understanding of material-process interactions.

### Mechanical Properties of 3D Printed Films and Electrospun Meshes

For reference, previously published data evaluated the mechanical performance of both electrospun meshes and 3D printed films at different blend ratios (95:5, 90:10, and 85:15).^14^ The results are summarized in Table 5 and illustrated in Figure 3f, demonstrating significant differences in both stress and strain (calculated using eqs 6 and 7, respectively) responses depending on blend composition and fabrication method. Notably, the 95:5 blend (red) demonstrated high strain, exceeding 724% in films and 548% in meshes, while the 90:10 blend (black) showed a marked reduction in strain (<452%) and a notable decrease in film strength (∼7.76 MPa). The 85:15 blend (blue) displayed divergent behavior, where films reached 685% strain, and meshes only achieved 278%; strength values for this blend also differed between formats, with films at ∼4.5 MPa and meshes at ∼3.3 MPa.

**Table 5:** Mechanical Properties of 3D-Printed Films and Electrospun Meshes Prepared with Different PLLA-*co*-CL/PDLA Blend Ratios (95:5, 90:10, and 85:15), Including Ultimate Strain (%) and Ultimate Stress (MPa)

sample	ultimate strain (%)	ultimate stress (MPa)	Young’s modulus [E] (MPa)
95:5 Film	724 ± 66	25.4 ± 7.0	19 ± 3.9
90:10 Film	452 ± 100	7.76 ± 0.7	22.6 ± 3.4
85:15 Film	685 ± 41	24 ± 3.7	34.3 ± 6.4
95:5 Mesh	548 ± 49	4.54 ± 0.7	1.11 ± 0.09
90:10 Mesh	135 ± 15	6.48 ± 1.7	2.93 ± 0.15
85:15 Mesh	278 ± 17	3.33 ± 0.4	4.92 ± 0.22

While a monotonic trend in mechanical performance might be expected with increasing PDLA content due to enhanced crystallinity and SC formation, the observed nonmonotonic behavior suggests a more complex interplay between blend composition and processing method. Differences in crystalline microstructure induced by electrospinning vs 3D printing, as well as potential phase separation or uneven SC formation at specific ratios, may contribute to the variability.^31^ These findings underscore that mechanical performance in PLLA-*co*-CL/PDLA-based systems is governed not only by composition but also by processing-driven morphology.

The mechanical properties of differently manufactured samples with varying blend ratios are comparable to a range of existing materials used as cardiac coverings, showing the vast adjustability of these materials depending on the required application. For instance, natural biomaterials like bovine pericardium exhibit an ultimate tensile strength of 5–15 MPa and a strain at failure of 10–35%, aligning with the strength of the 3D printed films.^32^ Similarly, medical-grade polyurethanes—commonly used in vascular grafts and occluder membranes—exhibit strengths ranging from 10–50 MPa and strain values up to 500%, closely resembling the flexibility observed in the 95:5 and 85:15 film blends.^33^ Electrospun meshes, with their lower stress values (below 3 MPa for the 90:10 blend and 4.9 MPa for the 85:15 blend), reflect mechanical behavior similar to PTFE/ePTFE, which exhibits a tensile strength of 10–20 MPa and a strain range of 10–20%.^34^

When compared to native cardiovascular tissues, the relevance of these mechanical profiles becomes even clearer. Human myocardium exhibits a Young’s modulus of approximately 0.02–0.5 MPa and ultimate strains exceeding 50%,^35^ while vascular tissues such as the aorta and coronary arteries typically show moduli of 0.1–1 MPa and strains of 60–120%.^36,37^ The electrospun meshes, with their low modulus and high extensibility (135–548%), therefore approximate the compliance and deformation capacity of soft cardiovascular tissues more closely than conventional nondegradable coverings such as ePTFE or PET, which are significantly stiffer (200–700 MPa) and exhibit limited strain (<20%).^38,39^ In contrast, the 3D-printed films provide higher tensile strength (7–25 MPa) and moduli up to ∼34 MPa, aligning with the mechanical performance of synthetic elastomers used in vascular grafts and occluder membranes.

This duality- soft, compliant meshes and stronger, more load-bearing films-highlights the versatility of the PLLA-*co*-CL/PDLA system. For the left atrial appendage occluder (LAAO) coverings, where materials must combine flexibility (to accommodate cardiac motion) with sufficient structural integrity (to resist cyclic loading and prevent tearing), the mechanical profiles observed here fall within the desirable range. The high extensibility of electrospun meshes may enable them to conform dynamically to the heart’s complex movements, minimizing mechanical mismatch and reducing the risk of tissue irritation. Meanwhile, the superior strength of 3D printed films could provide essential reinforcement in regions requiring durability or anchoring. Together, these comparisons underscore the translational potential of PLLA-*co*-CL/PDLA blends and demonstrate that their mechanical behavior can be tailored to match both native tissue mechanics and the performance envelope of clinically used cardiovascular polymers.

Taken together, the mechanical profile of the films and meshes, combined with their tunable SC content, point toward specific cardiovascular applications where elasticity, compliance, and controlled degradation are essential. The electrospun meshes, with their high extensibility, low modulus, and ECM-like structure architecture, are particularly well suited for flexible cardiac coverings, such as LAAO coverings, atrial or ventricular patches, or conformal epicardial wraps, where materials must accommodate continuous cyclic deformation without inducing mechanical mismatch. In contrast, the 3D-printed films, which exhibit higher tensile strength and greater structural integrity, align more closely with applications load-bearing support, such as reinforced occluder caps, valve-adjacent sealing membranes, or localized reinforcement zones in hybrid devices. When considered alongside the favorable endothelial responses and hemocompatibility observed in subsequent sections, these findings indicate that PLLA-*co*-CL/PDLA blends are more promising for biodegradable cardiac coverings and occluder membranes, where a balance of compliance, durability, and biological integration is critical for long-term performance.

### Surface Wettability and Sterilization Effects

To evaluate surface properties relevant to cell interactions and tissue integration, the wettability of both 3D-printed films and electrospun meshes was measured before and after sterilization with EtO. This analysis, shown in Figure S6 and Table S1, was conducted to determine the influence of EtO sterilization on surface hydrophilicity–an important factor in protein adsorption and subsequent cell adhesion.

Surface wettability analysis revealed distinct differences between films and meshes, as well as the influence of EtO sterilization. Films exhibited moderate hydrophilicity with advancing contact angles ranging from 76.5° to 99.4°, while meshes showed significantly lower wettability, with advancing angles exceeding 127° across all compositions. Constant angle hysteresis was also markedly higher in meshes, reaching up to 134.5°—indicating more pronounced surface roughness and potential Cassie–Baxter wetting behavior.^40^ EtO sterilization generally reduced hysteresis in both films and meshes, suggesting a modest smoothing effect or alteration in surface chemistry. Lower wettability and contact angle hysteresis, particularly seen in electrospun meshes, typically correlate with reduced protein adsorption uniformity and weaker initial cell attachment, as cells generally prefer moderately hydrophilic surfaces that promote stable focal adhesions. This is consistent with the established Berg limit, which posits that below an advancing contact angle of ∼60–65°, water molecules form strong interactions with the surface, discouraging replacement by proteins; in contrast, above this threshold, water bonding weakens, allowing easier displacement by proteins and promoting unspecific cell and protein attachment.^41^

This suggests that while the meshes provide topographical cues, their surface wettability may hinder early cell adhesion compared to films, which display more favorable (lower) advancing angles and hysteresis, especially poststerilization.

These findings align with previous studies reporting that electrospun meshes, due to their fibrous and porous architecture, typically exhibit higher apparent hydrophobicity and contact angle hysteresis compared to flat film counterparts, which is often attributed to air entrapment and the Cassie–Baxter wetting regime observed on nanostructured surfaces.^30,42^

### Evaluating Cell Interactions and Material Cyto-Compatibility

Given the intended biomedical application, particularly as cardiac coverings, it was essential to assess the compatibility of the materials in biological environments. Ensuring safe interaction with biological tissues while promoting integration and functional performance is critical. As ECs play a key role in mediating platelets’ binding to implant surfaces under dynamic blood flow and static conditions, the HUVEC model is widely used for such *in vitro* studies, providing a reliable platform to assess cell-material interactions under controlled, static conditions, which still offer valuable insights into endothelial behavior and compatibility in early biological assessments.^43–45^

### Indirect Cytotoxicity Assessment

To preliminarily evaluate the biological compatibility of the materials and ensure that any leachables from the polymers do not induce acute cytotoxic effects, indirect extract-based cytotoxicity tests were conducted using HUVECs.

The cytocompatibility of PLLA-*co*-CL and PDLA blends at varying weight ratios (95:5, 90:10, and 85:15) were initially assessed using extract-based (indirect) testing with HUVECs, a well-established *in vitro* model for ECs’ compatibility. After 48 h of exposure to polymer extracts, brightfield microscopy (Figure S7) revealed healthy HUVEC morphology with no signs of detachment or cell rounding, indicating the absence of acute cytotoxicity.

Quantitative assays were conducted to quantify cell membrane integrity and metabolic activity following extract exposure: LDH and MTS assays were employed alongside viability analysis, providing a multifaceted view of cellular health in response to each polymer blend. Automated cytotoxicity assays confirmed high cell viability (>97%) for all blends (Figure S8a). MTS assays revealed robust metabolic activity with the highest levels recorded for 90:10 films (79%) and 85:15 meshes (75%) (Figure 4a). LDH assays, which quantify LDH release as a marker of cell membrane damage, indicated minimal cytotoxicity (<20% for all samples), significantly lower than the positive control (Figure 4b). All measurements were normalized to a control group set at 100% on day 7. Together, these results demonstrate the excellent cyto-compatibility of the materials, particularly in electrospun form, with the 90:10 and 85:15 blends showing superior support for metabolic activity. The detailed significant differences after 48 h hours of cultivation for performing cytotoxicity assays are presented in Figure S8b–d.

**Figure 4:**
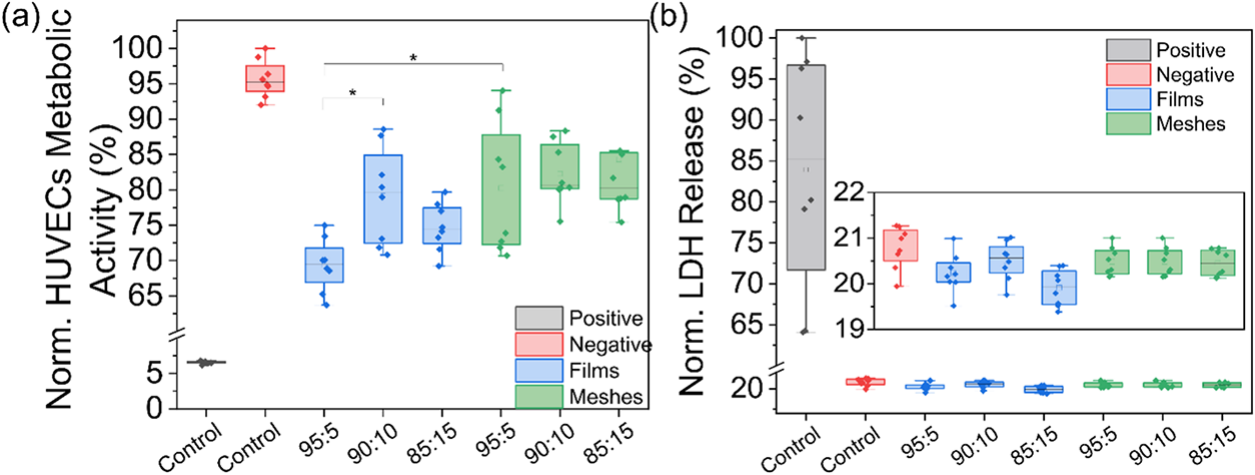
(a) Mitochondrial activity (normalized to the maximum value), and (b) lactate dehydrogenase (LDH) activity in the extracellular fluid (normalized to the maximum value) 48 h after culturing HUVEC cells with culture medium (negative control), with a 72 h-extract of the sample (1:0 and 1:10 dilution), and medium supplemented with Cell Lysis Solution (positive control). Values are given as arithmetic mean (bar) ± standard deviation (lines perpendicular to bar), *n* = 8. The inset in panel (b) shows an enlarged view of LDH activity, excluding the positive control, to better visualize group differences among the test and negative control conditions.

### Direct Cyto-Compatibility Studies

To further assess the cyto-compatibility under conditions more representative of *in vitro* contact, direct cell-material interaction studies were conducted, evaluating cell attachment, morphology, viability, and metabolic function over time.

Cell viability and metabolic activity (Figure 5a,c) were assessed directly on PLLA-*co*-CL/PDLA films and meshes across all blend ratios (95:5, 90:10, and 85:15) over a 7-day culture period, with assessments conducted on days 1, 3, and 7. Fluorescence images of live/dead HUVECs (representative images on 95:5 films and meshes taken on days 1, 3, and 7) are shown in Figure 5b. Additionally, actin staining fluorescence representative-images of HUVECs on 95:5 films and meshes on days 1, 7, and 14 are presented in Figure 5d.

**Figure 5:**
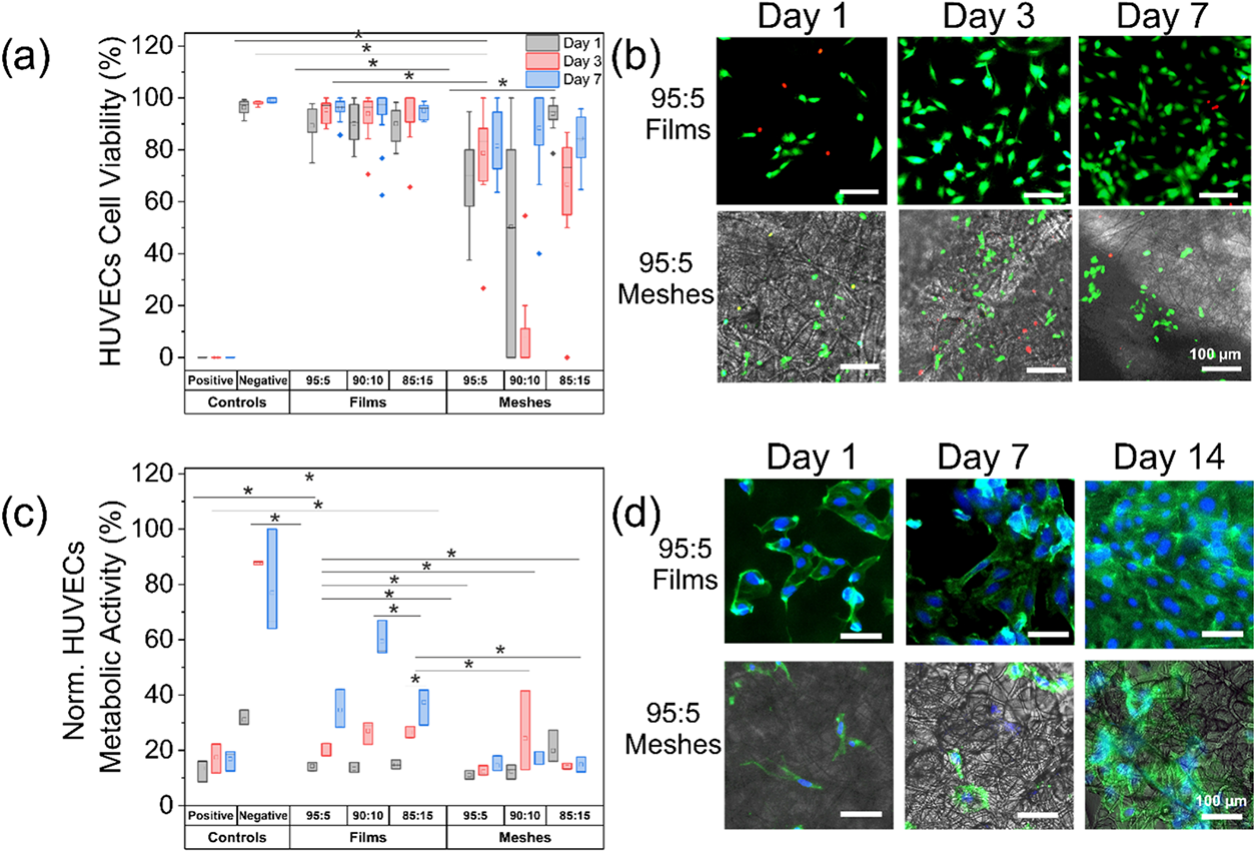
(a) Quantification of cell viability (%) based on the ratio of live cells to the total number of cells detected in the samples. (b) Representative fluorescence microscopy images (20× magnification) showing live (green) and dead (red) HUVECs cultured on films and meshes composed of PLLA-*co*-CL/PDLA at the 95:5 blend ratio over 1, 3, and 7 days. Scale bars: 100 μm. (c) Metabolic activity of HUVECs, measured as absorbance at 492 nm. (d) Fluorescence microscopy images of HUVECs cultured on films and meshes for 1, 7, and 14 days. Actin filaments are stained green, and cell nuclei are stained blue, highlighting cytoskeletal organization and cell distribution. For clarity in visualization, images of the films were contrast-adjusted (dark background) to better highlight stained cells; cells in both film and mesh groups were directly seeded on the material surface. Scale bar: 100 μm. Data are presented as mean ± standard deviation, with statistical significance denoted by * (*p* ≤ 0.05).

Figure 5a shows a steady increase in cell viability for all film samples from day 1 to 3 and 7, demonstrating a viability greater than 94% by day 7. The highest viability was observed on the 95:5 film, where viability increased from 89.3% on day 1 to 96.2% on day 7. Similar trends were observed for the 90:10 and 85:15 films. As shown in Figure 5b, this increasing trend on films is also visually evident, with a progressive rise in the number of viable (green) cells from day 1 to 7. In contrast, the behavior of the meshes was different, where the 95:5 meshes displayed an increase in viability from 67.8% on day 1 to 81.5% on day 7 (similar to the films) while the 90:10 and 85:15 meshes exhibited a decrease in viability between days 1 and 3, followed by recovery by day 7. These distinct trends are also reflected in the fluorescence images in Figure 5b, which illustrates the differing viability patterns between films and meshes. Viability images of HUVECs on the 90:10 and 85:15 blend ratios are shown in Figure S9. The highest viability among the meshes was observed on the 90:10 blend, with a viability of 88.4% on day 7.

A similar trend was observed in metabolic activity using the MTS assay (Figure 5c), which assesses the metabolic health and activity of HUVECs by measuring the reduction of MTS to formazan, a product formed by mitochondrial enzymes in viable cells. On both films and meshes, an increase in metabolic activity was noted over the 7-day culture period, with the highest metabolic activity recorded on the 90:10 films at day 7 (67%). This increase in metabolic activity aligns with the increase in cell viability observed for films, where consistent cell proliferation and viability were observed over time.

The 95:5 meshes exhibited a steady increase in metabolic activity, similar to the films, with values rising from day 1 to day 7, reflecting improved cell attachment and proliferation on the mesh surface. However, the behavior of meshes with other blend ratios (90:10 and 85:15) was distinct. The 90:10 meshes showed a decrease in metabolic activity from day 3 (41%) to day 7 (21%), and 85:15 meshes exhibited a decline in metabolic activity from day 1 (16%) to day 3 (13%), followed by a slight increase on day 7 (15%). These fluctuations may reflect differences in surface morphology and early cell-material interactions that temporarily affect cell function or attachment. Importantly, indirect cytotoxicity testing confirmed the absence of toxic leachables (e.g., solvent residues (Figure 4)), excluding cytotoxicity as a potential cause of reduced metabolic activity.

These observations are in line with the surface wettability results (Figure S6 and Table S1), which showed that electrospun meshes had significantly higher contact angles and hysteresis compared to films, indicating increased hydrophobicity and surface roughness. While such surfaces are generally associated with higher levels of unspecific protein and cell adhesion due to their hydrophobic nature- particularly above the Berg limit of ∼60–65°—they may also lead to nonselective interactions and hinder optimal integrin-mediated adhesion and cell spreading. This complex relationship between hydrophobicity, topography, and biological response has been highlighted in previous studies.^41,46^

This reflects the widely accepted model in which advancing contact angles above 65° enable water displacement, facilitating protein adsorption through unspecific mechanisms, while lower angles tend to prevent such displacement. However, although high contact angles can increase general protein adhesion, this does not always promote specific or functional cell adhesion, especially in the absence of integrin-binding motifs or favorable mechanical cues.

Overall, these findings suggest that while hydrophobic, certain topographies of nontreated electrospun meshes may promote nonspecific interactions and compromise early organized cell attachment and metabolic function- particularly in specific blend compositions.

Given that the average pore size (∼5.1 μm) and fiber dimensions fall within a range that permits cellular interaction (though not full infiltration), the divergence in metabolic behavior is more likely attributed to surface morphology and material-specific interaction, rather than pore inaccessibility or cytotoxicity. In some blends, insufficient scaffold stiffness or local fiber instability may initially impair stable cell anchorage, leading to transient reductions in metabolic activity that likely reflect early cellular stress before subsequent adaptation.

Fluorescence microscopy after DAPI/Phalloidin staining (Figures 5d and S10) showed increased cell density over time, with more pronounced coverage on film surfaces compared to meshes. DAPI stains the cell nuclei (blue), allowing the visualization of cell density and distribution, while Phalloidin selectively binds to the F-actin filaments (green), highlighting the cytoskeletal organization and cell morphology. Over the 14-day culture period, both cell number and spreading increased, shown in Figure 5d, indicating enhanced cell attachment and proliferation relative to the more topographically complex and less hydrophilic mesh surfaces.

The difference in metabolic activity and viability between films and meshes may be explained by the distinct structural characteristics of these materials. Films, with their continuous, smooth surface, likely provide a more favorable environment for HUVECs attachment and proliferation, contributing to the steady increase in both cell viability and metabolic activity. In contrast, meshes, with their porous and less uniform structure, lead to a more complex cellular response, particularly seen on the 90:10 and 85:15 blend ratios, where a decrease in metabolic activity was observed at intermediate time points. This disparity can also be associated with the differences in surface wettability. Electrospun meshes exhibited significantly higher advancing contact angles and hysteresis compared to films, indicating greater surface hydrophobicity and roughness. Such properties are known to impair uniform protein adsorption and reduce initial cell adhesion, likely contributing to the delayed or suppressed metabolic activity and viability observed on mesh substrates.

This behavior is somewhat unexpected, as mesh architecture typically promotes better cell attachment due to increased surface area and topographical cues, which were seen with the actin staining. However, SEM analysis revealed that although the meshes had a pore size averaging 5.1 μm and fiber diameter around 3–4 μm (as seen in Figures 3d,e, and S4)–dimensions theoretically compatible with cellular interaction—cells did not infiltrate the scaffold significantly. This limited infiltration aligns with literature reports indicating that pore sizes smaller than the approximate diameter of endothelial cells (∼17 μm) restrict cell penetration and limit growth primarily to the scaffold surface.^47,48^ While pores around 5 μm can support cell spreading and attachment, they are generally insufficient to enable full cellular infiltration, which is critical for 3D tissue formation and sustained metabolic activity. Since the MTS assay reflects metabolic activity throughout the entire scaffold, this suggests that the lower signal is not due to hidden or internalized cells, but rather likely due to a reduction in surface-attached viable cells. Notably, the majority of studies employing electrospun meshes for endothelial culture report the use of additional surface modifications–such as plasma treatment or protein coatings–to improve wettability and bioactivity. Incorporating such strategies may therefore be recommended in future applications to mitigate limitations arising from intrinsic surface hydrophobicity and restricted pore accessibility.

Several factors may account for the observed differences in cellular response between films and meshes. Unlike 3D printed films, electrospun meshes typically exhibit lower stiffness (Young’s modulus) and more heterogeneous surface topography, especially at certain blend ratios. These mechanical and structural features can significantly influence ECs’ behavior, which is highly sensitive to substrate mechanics. ECs tend to favor moderately stiff environments (∼1–10 kPa) that promote cytoskeletal organization, focal adhesion formation, and metabolic activity.^49^ If the local stiffness of the mesh falls below this physiological range, or is highly variable due to uneven fiber distribution, it may impair early cell attachment and spreading. Additionally, the irregular morphology of meshes can hinder uniform protein adsorption, which is critical for integrin-mediated adhesion and subsequent cell survival. Even when composed of the same material blends, differences in fabrication methods introduce variations in surface roughness, wettability, and polymer chain orientation, all of which shape the protein adsorption landscape and integrin engagement. These factors directly influence cytoskeletal organization, as reflected in the actin staining results (Figure S9), where more extensive F-actin filament formation and increased cell spreading were observed on films compared to the meshes. The higher confluency and more organized actin structures on films enhanced integrin-mediated adhesion and mechano-transduction, likely due to their more favorable surface characteristics.^50,51^ The initial decline in metabolic activity in 90:10 and 85:15 meshes may reflect early cell stress or insufficient attachment cues, with partial recovery observed by day 7 in the 85:15 group, suggesting eventual adaptation.

These findings indicate that while the mesh architecture provides structural advantages, its effectiveness is highly dependent on blend and topography-specific properties and how these affect the mechanical, chemical, and topographical signals perceived by the cells. Overall, these findings demonstrate that the blend ratio and material morphology play a significant role in influencing both cell viability and metabolic activity, with films generally supporting more consistent cellular growth and metabolic health, while meshes exhibit more variability.

The findings suggest that PLLA-*co*-CL/PDLA blends can be used in vascular or cardiac device coverings, where compatibility and interaction with endothelial cells are crucial. Further assessment of vasoactive substance release underscores the functional compatibility of these materials with endothelial cell activity.

### Evaluation of Vascular Tone by Endothelial Cells Cultured on Polymeric Substrates

Given the intended vascular and cardiac applications of these biomaterials, assessing the functionality of ECs in contact with the polymer surfaces is essential. Beyond viability, functional EC behavior includes the regulated production of vasoactive substances, which play critical roles in maintaining vascular homeostasis. Three key markers were selected to assess EC activity: nitric oxide (NO), prostacyclin (PGI_2_), and thromboxane B_2_ (TXB_2_).

In parallel with endothelial functional assessment, preliminary blood-material interactions were evaluated using a direct-contact hemolysis assay in accordance with ISO 10993–4 principles as an initial *in vitro* screening (Figure 6a), which evaluates the extent to which a biomaterial surface induces RBC membrane damage and subsequent hemoglobin release.

**Figure 6:**
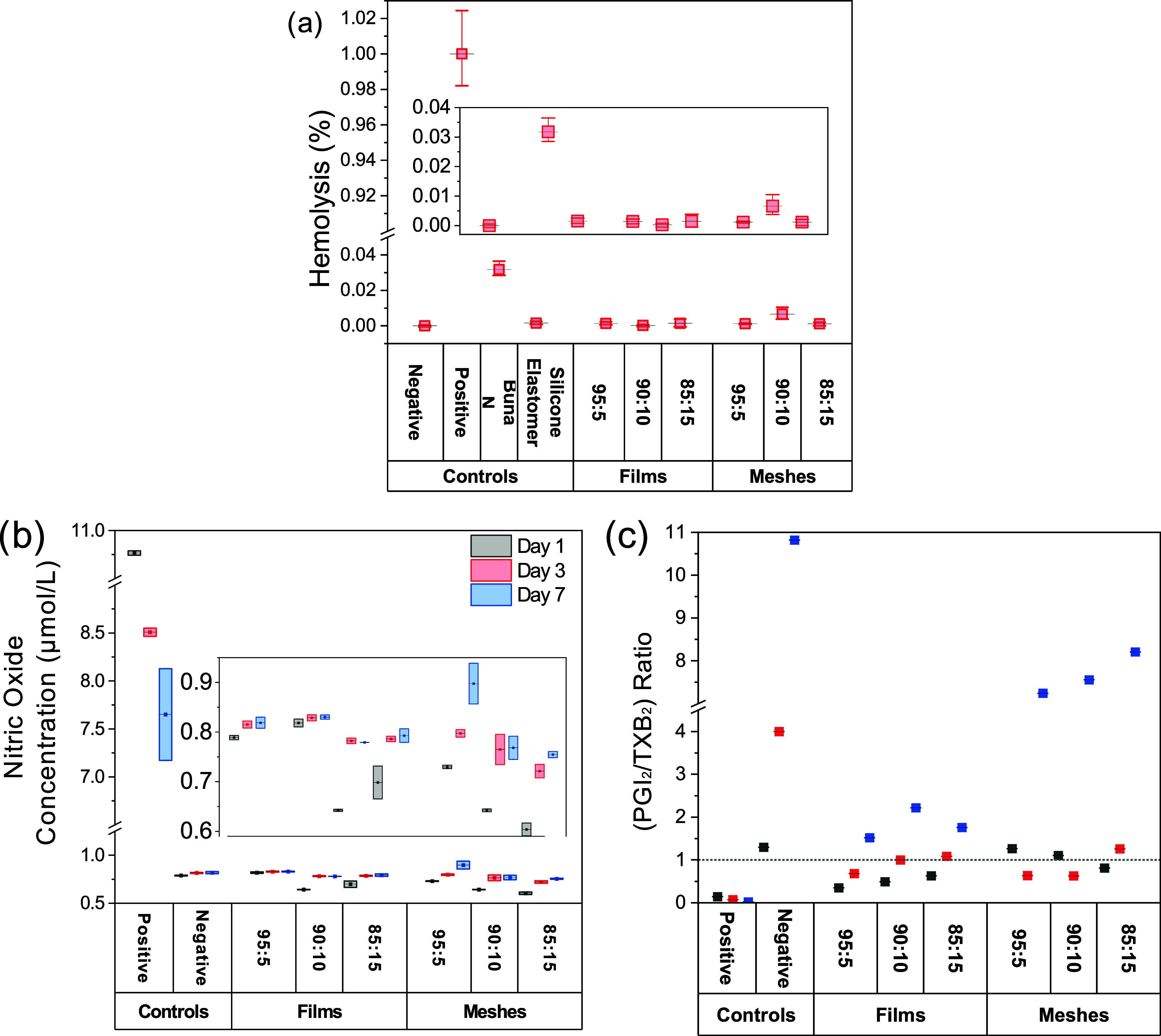
(a) Direct-contact hemolysis assay results showing all tested films and meshes, as determined by direct incubation for 24 h in erythrocyte suspension and subsequent determination of free hemoglobin in sample supernatant. The inset in (a) excludes the positive control data to improve visualization of differences among experimental groups. Secretion of vasoactive substances by HUVECs cultured on scaffolds with a density of 20,000 cells/well for a period of 7 days. The total concentrations of (b) nitric oxide (NO) released (expressed as μmol/L), quantified using ELISA. The inset in (b) excludes the positive control data to improve visualization of differences among experimental groups. (c) The ratio of prostacyclin to thromboxane B_2_ (TXB_2_) was calculated, where a ratio of 1 reflects a balanced/healthy endothelial state, a ratio <1 indicates a pro-thrombotic state, and a ratio >1 indicates an antithrombotic state. Statistical significance was determined by ANOVA (*p* ≤ 0.05), with significant differences indicated by (*).

Appropriate controls were included to benchmark assay performance. Erythrocyte suspension in PBS with no samples added served as the negative control, representing baseline absorbance associated with spontaneous hemolysis, while erythrocyte suspension diluted 1:10 in DI was used as the positive control, representing complete hemolysis. Additionally, silicone elastomer reference material was included as a clinically relevant, nonhemolytic biomaterial control, along with Buna-N (nitrile rubber), a material known to induce limited RBC lysis, which served as a positive hemolytic material control in accordance with ISO 10993–4. As expected, the positive hemolytic controls substantially elevated hemolysis values (calculated according to eq 8), with Buna-N (3.177%) and DI water (100%, no visible erythrocyte pellet observable following centrifugation postincubation) confirming the sensitivity and dynamic range of the assay.

While minor numerical differences were observed among blend ratios and between films and meshes, these variations remain within the nonhemolytic range (below 2%, as outlined per ISO 10993–4) and should not be overinterpreted as biologically meaningful differences in blood-material interactions. The hemolysis assay employed here is intended as an initial screening tool to identify overt RBC membrane disruption, rather than to resolve subtle differences in overall blood compatibility. Accordingly, the data demonstrate that all polymer compositions, as both films and mesh formats, evaluated here are nonhemolytic and suitable for further investigation.

Taken together, these results provide preliminary data on erythrocyte-material interactions that complement the endothelial functional results presented in this study. More comprehensive evaluations of blood-material interactions, including platelet activation and coagulation-related pathways, will be required in future work to fully characterize thrombogenic potential.

NO is a vasodilator produced by endothelial nitric oxide synthase and is essential for inhibiting platelet aggregation, smooth muscle cell proliferation, and inflammation.^52^ PGI_2_ similarly promotes vasodilation and prevents platelet activation, while TXB_2_—a metabolite of thromboxane A_2_—is a potent vasoconstrictor and pro-thrombotic molecule. Together, the balance of these molecules serves as a sensitive indicator of endothelial health and vascular compatibility.^53,54^

The production of vasoactive substances (represented in Figures 6b,c and S11) is a characteristic property of functional endothelial cells. The release of NO, as seen in Figure 6b, increases from day 1 to day 7 on all films and meshes (at all blend ratios). This indicates that across all compositions and formats, endothelial function is supported. The reduced NO release, as seen with the positive control, indicates endothelial dysfunction and is a hallmark of atherosclerosis and thrombosis. Surpassing the negative control values (∼0.85 μmol/L), the highest NO levels were obtained on 95:5 meshes and films, of 0.90 and 0.82 μmol/L, respectively, suggesting that this composition enhances endothelial activity beyond normal baseline.

Besides NO, we have measured the release of PGI_2_ and TXB_2_—key markers of antithrombotic and pro-thrombotic activity of endothelial function, respectively—after incubation of cells with various samples for a period of 7 days (Figure S11a,b). Figure S11a shows a consistent upward trend across all tested substrates. The highest levels were observed on films, with the 90:10 blend reaching approximately 966 pg/mL by day 7. Meshes also showed significant PGI_2_ release, peaking for the 85:15 blend (∼696 pg/mL), though overall release from meshes remained lower than that of films. These results suggest that films provide a more stable and supportive environment for sustained endothelial function, while meshes may initially promote activity but with more fluctuation.

In comparison, PGI_2_ release was notably low in the positive control, reflected in the progressive decline reaching values below 20 pg/mL. In contrast, in the negative control, PGI_2_ release increased steadily from day 1 to 7, reaching 31 pg/mL, suggesting that under normal culture conditions, HUVECs maintain a stable capacity to produce PGI_2_, with an increasing trend likely due to cell proliferation and enhanced metabolic activity over time.

As shown in Figure S11b, the positive control group, treated with Tumor necrosis factor α (TNF-α), decreases progressively from day 1 to day 7. TNF-α suppresses PGI_2_ production by endothelial cells, reducing their anti-inflammatory and vasodilatory effects, while often promoting an increase in TXB_2_ levels, which encourages vasoconstriction and platelet aggregation, and can decrease NO bioavailability, further impairing vascular relaxation and enhancing inflammation.^55,56^ This aligns with the known effects of TNF-α in promoting endothelial dysfunction, leading to impaired eicosanoid metabolism and reduced thromboxane synthesis over time.^55–57^

In contrast, the negative control showed a steady increase in TXB_2_ levels, reaching 1200 pg/mL by day 7, suggesting that under unstimulated conditions, endothelial cells maintain an active TXB_2_ production pathway. In contrast, TXB_2_ release (Figure S11b) on films increased markedly by day 7 across all blends, with the 95:5 film reaching ∼500 pg/mL, suggesting improved cell maturation and pro-thrombotic potential. However, meshes displayed an initial spike in TXB_2_ (especially the 85:15 mesh with ∼252 pg/mL on day 1), followed by a sharp decline by day 7, potentially reflecting early stress followed by adaptation or decreased cell activity.

The release patterns of PGI_2_ and TXB_2_ suggest that the materials actively engaged endothelial arachidonic acid metabolism over time. Both films and meshes simulate the release of these vasoactive mediators, indicating that the materials are not inert, but rather interact dynamically with ECs. However, the kinetics and magnitude of this activation differ depending on the material format.

In films, the release of both PGI_2_ and TXB_2_ increased over a 7-day culture period (Figure S11), but the increase in PGI_2_ was significantly more pronounced than that of TXB_2_. This disproportionate rise suggests a dominant antithrombotic response, where ECs, although activated, favor a protective phenotype. Such a pattern is consistent with compensatory counter-regulation, in which the endothelium attempts to maintain vascular homeostasis by enhancing PGI_2_ production to offset concurrent thromboxane signaling. The sustained TXB_2_ production, however, indicates that the pro-thrombotic pathway is also engaged and remains active throughout, though it does not predominate. This dual activity supports the interpretation that films continuously activate ECs, prompting a protective, yet attentive state, a response that could be beneficial in cardiovascular implants requiring both hemocompatibility and rapid endothelial integration.

In meshes, the behavior follows a distinct trajectory. While an initial spike in TXB_2_ release is observed–particularly notable for the 85:15 mesh on day 1, a sharp decline in TXB_2_ by day 3 is evident, followed by a rebound in PGI_2_ release through day 7. This pattern differs from that of films and may reflect a more physiological and adaptive response of the ECs to an initial external stimulus. The early TXB_2_ spike could represent a transient activation phase, as ECs respond to the complex topography and higher surface roughness of the meshes. The subsequent decline in TXB_2_ and sustained PGI_2_ production suggests that cells adapt to the mesh microenvironment over time and recover a more stable, antithrombotic phenotype. Such kinetics have been reported in other studies where ECs exhibit similar responses to new substrates or even after routine medium changes, underscoring this as a ‘normal’ adaptation to environmental perturbation.^58,59^

To better interpret these trends, the PGI_2_/TXB_2_ ratio over 7 days was calculated (Figure 6c). A ratio >1 is considered indicative of a functional, antithrombotic endothelial phenotype dominated by PGI_2_ activity, a ratio = 1 suggests a balanced and homeostatic endothelial state, and a ratio <1 reflects a shift toward a pro-thrombotic, activated, or dysfunctional state where TXB_2_ predominates. In the positive control, this ratio remained below 1 throughout the culture period, confirming persistent dysfunction. In contrast, the negative control maintained a ratio >1 from day 1 to 7, supporting stable endothelial function.

On the films, the PGI_2_/TXB_2_ ratio increased from below 1 on day 1 to above 1 by day 7 for all blend ratios, demonstrating a progressive shift toward an antithrombotic phenotype. This further supports the idea of ongoing endothelial activation by the films, but with protective dominance of prostacyclin signaling, consistent with the elevated PGI_2_ levels and sustained cell viability and metabolic activity.

On the meshes, the ratio started above 1 on day 1, dropped below 1 by day 3, and rebounded by day 7. This dynamic profile suggests an initial stress response–possibly due to structural or surface-related cues—followed by cellular adaptation and recovery. The early PGI_2_ production and transient TXB_2_ elevation, followed by stabilization, further indicates that meshes provoke a more acute but ultimately normalized endothelial response, reflective of an initial activation phase and subsequent return to a protective state.

The differences between films and meshes can be attributed to their distinct structural and surface characteristics, resulting from the fabrication method. Films, being flat and continuous, offer a uniform surface with lower roughness and more moderate wettability, facilitating consistent protein adsorption and stable integrin engagement, which supports progressive endothelial activation and protective signaling. In contrast, electrospun meshes are highly porous, topographically complex, and more hydrophobic, which initially challenges cell adhesion and induces stress-related responses, as evidenced by the transient drop in the PGI_2_/TXB_2_ ratio. However, the greater available surface area and 3D architecture of the meshes may also promote more extensive cell-material interaction over time, ultimately supporting endothelial recovery.

Among the tested ratios, the 90:10 blend demonstrated the most balanced and favorable performance, with high cell viability (94.5%), robust metabolic activity (67% on day 7), and the highest PGI_2_ release (966 pg/mL) by day 7, resulting in a PGI_2_/TXB_2_ ratio above 1 that is sustained through day 7. This composition supports a stable, antithrombotic, and endothelial-compatible environment, making it particularly promising for implantable cardiovascular devices where long-term endothelial function and thromboresistance are critical.

In contrast, 85:15 meshes, while showing early activation, had elevated TXB_2_ release (252 pg/mL) and more variable PGI_2_ output, suggesting a less favorable, pro-thrombotic tendency, potentially limiting their utility in blood-contacting applications.

## Conclusion

This work demonstrates that PLLA-*co*-CL/PDLA blends form a versatile, tunable material platform with mechanical, thermal, and biological properties well suited for cardiovascular implant coverings. By tailoring PDLA content and exploiting SC formation, the blends achieve a balance of elasticity, strength, and controlled degradation that cannot be obtained from single-polymer systems. Processing the same compositions to electrospun meshes and 3D printed films further revealed how fabrication-driven morphology modulates compliance and endothelial response.

Among the tested formulations, the 90:10 PLLA-*co*-CL/PDLA blend struck the optimal balance, providing sufficient mechanical integrity, endothelial viability, and biofunctionality, including high PGI_2_ and low TXB_2_ release—indicative of a nonthrombogenic, vaso-protective phenotype. All materials exhibited a nonhemolytic behavior, supporting their suitability for blood-contacting environments.

In summary, these findings identify biodegradable cardiac coverings–particularly LAAO coverings and related flexible sealing structures–as the most promising application for this material system. The blend’s compliance, endothelial compatibility, and tunable SC reinforcement position them as strong candidates to complement or replace current nondegradable coverings. While further *ex vivo* and *in vivo* studies are required, this study establishes a clear foundation for advancing PLLA-*co*-CL/PDLA blends toward next-generation bioactive cardiovascular implants.

## Supplementary Material



## Notes

^13^C NMR spectra of PLLA-*co*-CL and PDLA; GPC chromatograms and molecular-weight analyses; derivative TGA profiles of synthesized polymers; optimization details for 3D printing and electrospinning parameters; representative images of optimized constructs (film and mesh); rheological data for 90:10 and 85:15 blends; contact angle measurements; brightfield images of HUVECs with indirect cytotoxicity assay results; fluorescence microscopy images showing live/dead staining, actin filaments, and nuclei; quantitative measurements of prostacyclin (PGI_2_) and thromboxane (TXB_2_) release. 1. ^13^C NMR spectra (Figure S1) 2. GPC analyses (Figure S2a) 3. dTGA profiles (Figure S2b) 4. Rheological data (90:10, 85:15) (Figure S3) 5. 3D printing optimization details (Figure S4a,b) 6. Electrospinning optimization details (Figure S4c,d) 7. Representative images of optimized constructs: extrusion-printed film and electrospun mesh (Figure S5) 8. Contact angle measurements (Figure S6 and Table S1) 9. Brightfield HUVEC images + cytotoxicity (Figures S7 and S8) 10. Fluorescence microscopy images (live/dead) (Figure S9) 11. Fluorescence microscopy images (Confluency) (actin filaments and cell nuclei) (Figure S10) 12. PGI_2_ and TXB_2_ quantitative data (Figure S11) (PDF)
