# Supplementary material for: 3D Printing and Electrospinning of PLLA-co-CL/PDLA Blends as Potential Materials for Cardiovascular Implants
Source: ACS Biomater Sci Eng. 2026 Apr 28;12(5):2648–66. doi: 10.1021/acsbiomaterials.6c00151 (PMC13169387; doi:10.1021/acsbiomaterials.6c00151)
Supplement: Supplementary file 1 [file ab6c00151_si_001.pdf]

# 3D Printing and Electrospinning of PLLA-*co*-CL/PDLA Blends as Potential Materials for Cardiovascular Implants

*Hanin Alkhamis[1], Angelika Ritschel[2], Lennard K. Shopperly[3], Sahar Salehi[4, 5], Axel T. Neffe[6], Katarzyna Polak-Kraśna[1,*]*

[1] Institute of Active Polymers, Helmholtz-Zentrum Hereon, Kantstraße 55, 14513 Teltow, Germany

[2] Institute of Functional Materials for Sustainability, Helmholtz-Zentrum Hereon, Kantstraße 55, 14513 Teltow, Germany

[3] Charité – Universitätsmedizin Berlin, Corporate Member of Freie Universität Berlin and Humboldt-Universität zu Berlin, Centre for Trauma- and Reconstructive Surgery, Hindenburgdamm 30, 12203 Berlin, Germany

[4] Professor for Engineering Biointelligent System, Institute of Food Science and Biotechnology, University of Hohenheim, Garbenstrasse 25, 70599, Stuttgart, Germany

[5] Fraunhofer Institute for Manufacturing Engineering and Automation (IPA), Nobelstrasse 12, 70569 Stuttgart, Germany

[6] Institute of Materials Chemistry, BTU Cottbus-Senftenberg, Universitätsplatz 1, 01968 Senftenberg, Germany

* Katarzyna.Polak-Krasna@hereon.de

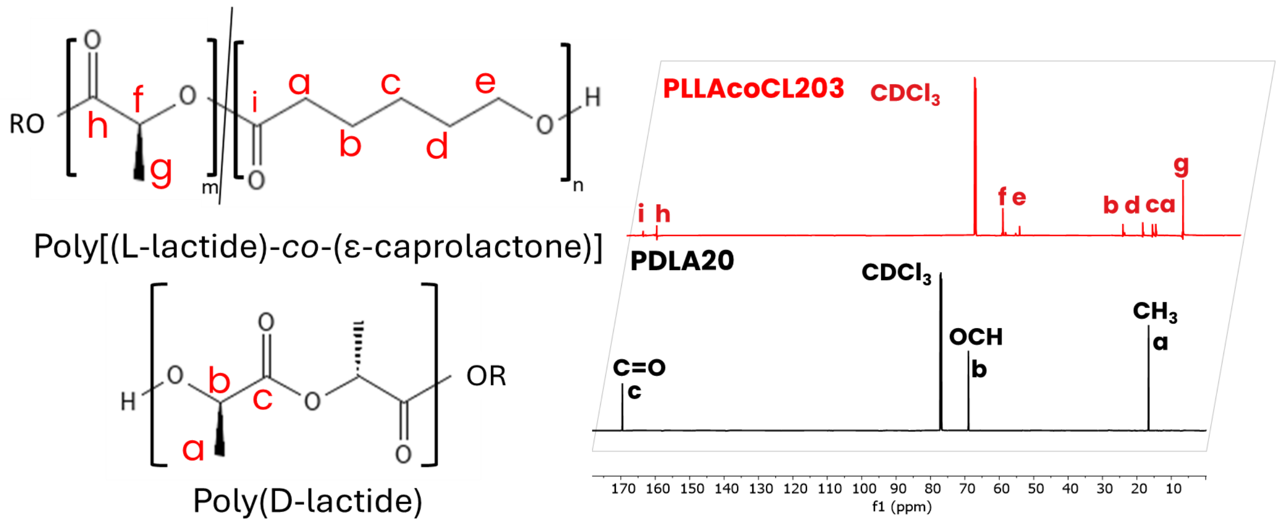


Figure S 1: 13C-NMR spectra of PLLA-*co*-CL of 203,000 g mol-1 (red), and PDLA of 20,000 g mol-1 (black).

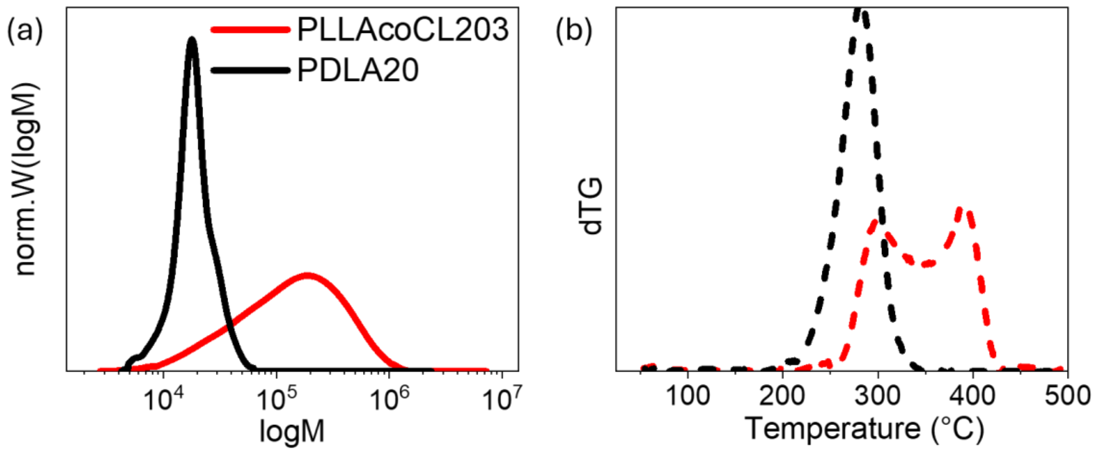


Figure S 2: a) GPC Profile and b) dTG (obtained via TGA) of PLLA-*co*-CL of 203,000 g mol-1 (red), and PDLA of 20,000 g mol-1 (black).

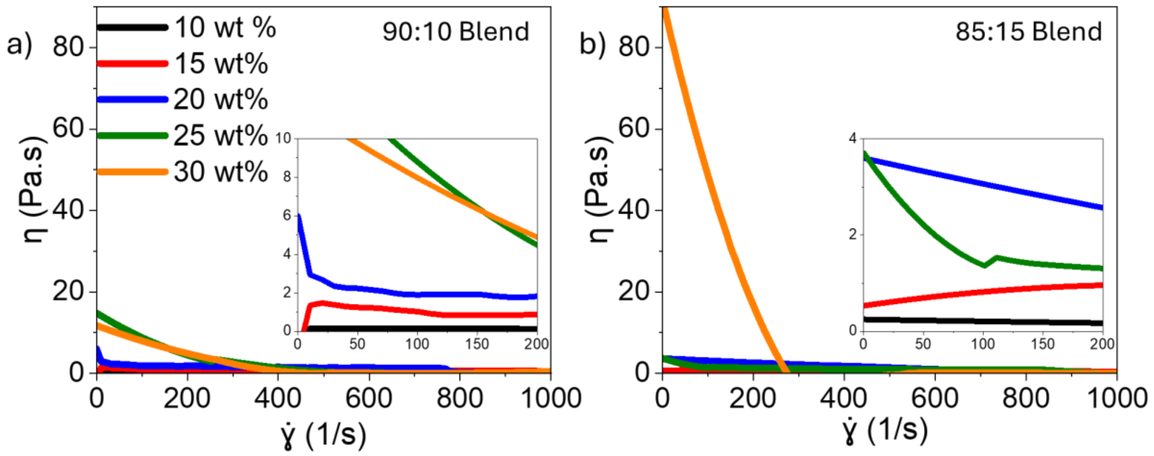


Figure S 3: Rheological properties of solutions with varying concentrations (10, 15, 20, 25, and 30 wt%) of (a) 90:10 and (b) 85:15 PLLA-*co*-CL/PDLA blend in chloroform. The inset displays the viscosity response in the lower shear rate range (0–200 $s^{-1}$) for improved visualization of differences at low deformation rates.

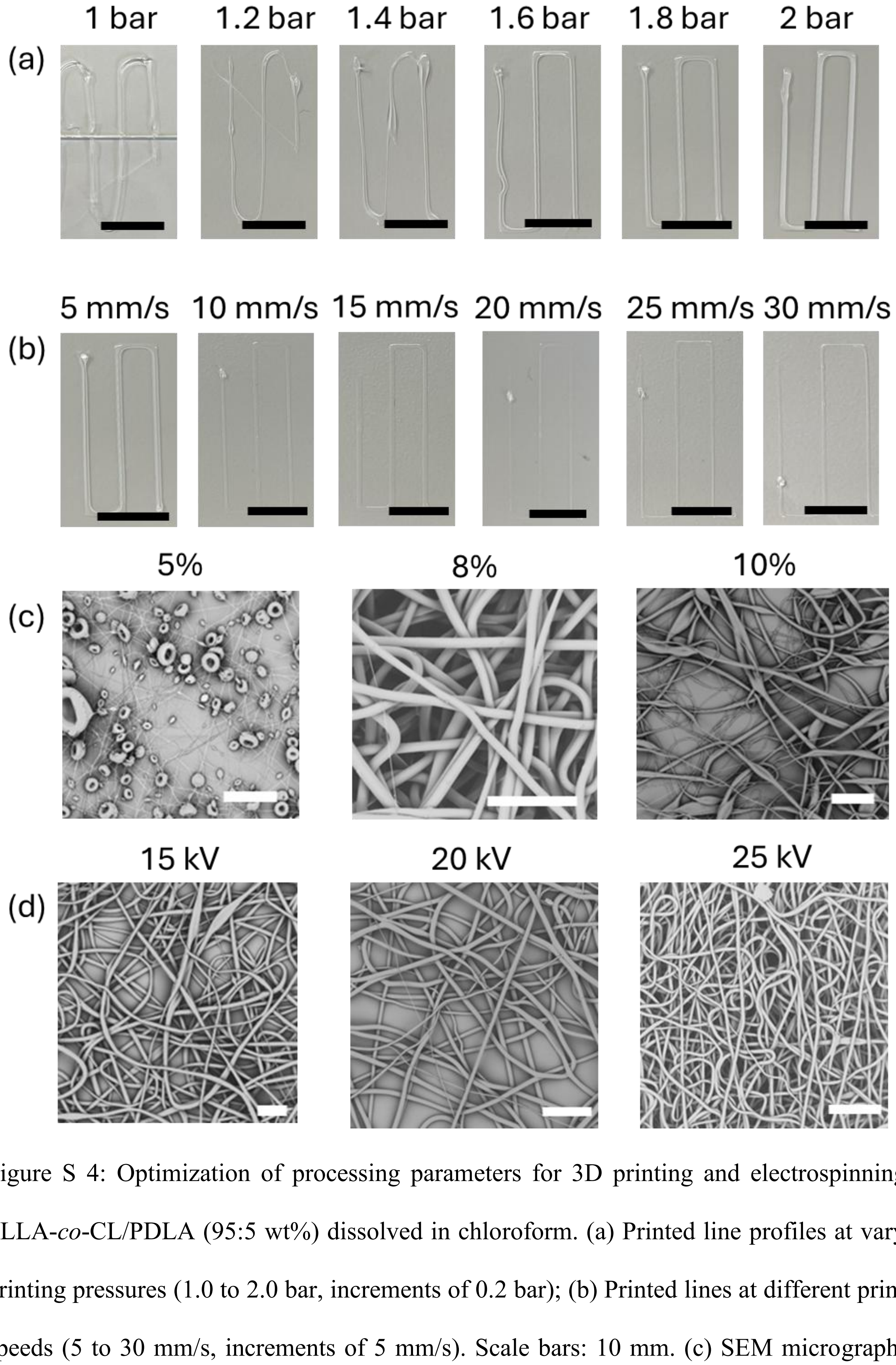


Figure S 4: Optimization of processing parameters for 3D printing and electrospinning of PLLA-*co*-CL/PDLA (95:5 wt%) dissolved in chloroform. (a) Printed line profiles at varying printing pressures (1.0 to 2.0 bar, increments of 0.2 bar); (b) Printed lines at different printing speeds (5 to 30 mm/s, increments of 5 mm/s). Scale bars: 10 mm. (c) SEM micrographs of electrospun meshes obtained using varying polymer solution concentrations (5%, 8%, and 10%

w/v). Scale bars: 40 µm. (d) SEM images of fibers electrospun under different applied voltages (15, 20, and 25 kV). Scale bars: 10 µm.

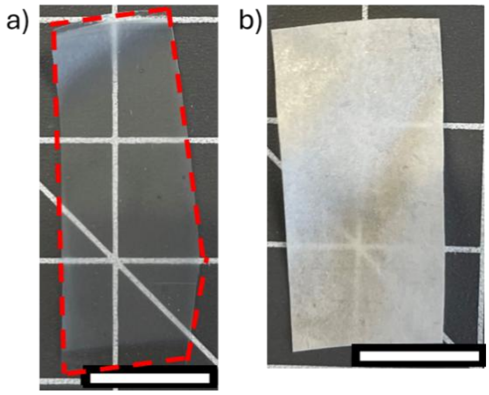


Figure S 5: Representative images of the optimized constructs: (a) extrusion-based 3D printed film (outlined by dashed red line) and (b) electrospun mesh. These morphologies reflect the final outcomes of the processing parameter optimization. Scale bar: 10 mm.

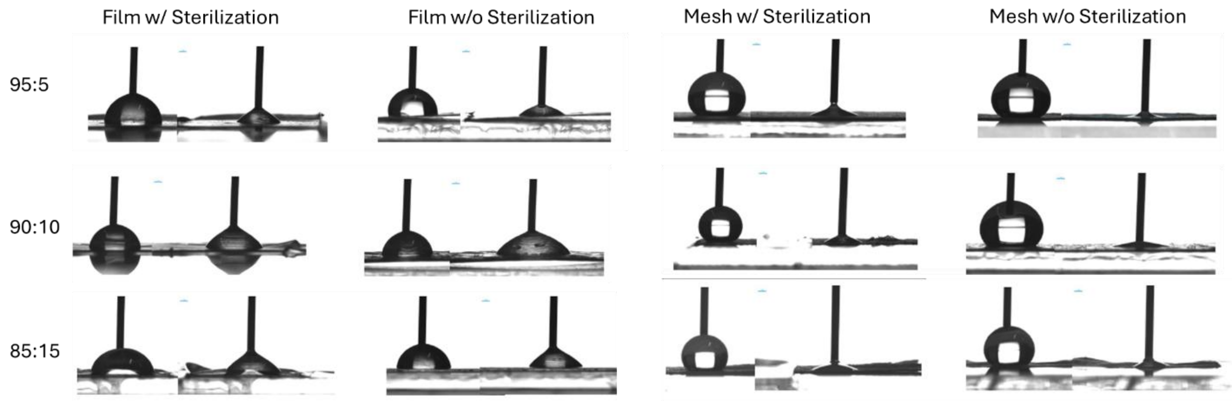


Figure S 6: Contact angle images of water droplets on films and meshes, obtained using the sessile drop method at (1)22°C with water-air media. Advancing and receding angles were measured to evaluate surface wettability prior to cell culture experiments, investigating the effect of ethylene oxide sterilization on cell adhesion. The films and meshes were processed via 3D printing and electrospinning using 20 wt% and 8 wt% polymer solutions, respectively, with blend ratios of 95:5, 90:10, and 85:15 of PLLA-*co*-CL203:PDLA20.

Table S 1: Advancing, receding, and contact angle hysteresis measurements for films and meshes processed via 3D printing and electrospinning, with and without ethylene oxide sterilization.

| Sample (blend ratio, film/mesh) | Ethylene Oxide Sterilization (Yes/No) | Advancing ± Stdev. (°) | Receding ± Stdev. (°) | Hysteresis (Δ°) |
|---|---|---|---|---|
| 95:5 Film | Yes | 84.8 ± 2.1 | 46.1 ± 2.9 | 38.8 |
| 95:5 Film | No | 99.4 ± 6.3 | 38.5 ± 3.4 | 60.9 |
| 90:10 Film | Yes | 82.8 ± 2.4 | 52.7 ± 6.4 | 30.1 |
| 90:10 Film | No | 82.8 ± 2.0 | 54.6 ± 4.0 | 28.2 |
| 85:15 Film | Yes | 76.5 ± 4.0 | 51.8 ± 2.7 | 24.7 |
| 85:15 Film | No | 83.9 ± 1.9 | 52.5 ± 2.4 | 31.5 |
| 95:5 Mesh | Yes | 148.7 ± 1.8 | 14.2 ± 1.9 | 134.5 |
| 95:5 Mesh | No | 148.5 ± 4.6 | 20.0 ± 0.2 | 128.5 |
| 90:10 Mesh | Yes | 153.6 ± 3.8 | 20.4 ± 9.5 | 133.2 |
| 90:10 Mesh | No | 143.4 ± 2.1 | 12.4 ± 1.1 | 131.0 |
| 85:15 Mesh | Yes | 127.2 ± 2.4 | 20.1 ± 1.2 | 107.1 |
| 85:15 Mesh | No | 137.2 ± 4.6 | 22.2 ± 5.0 | 115.0 |

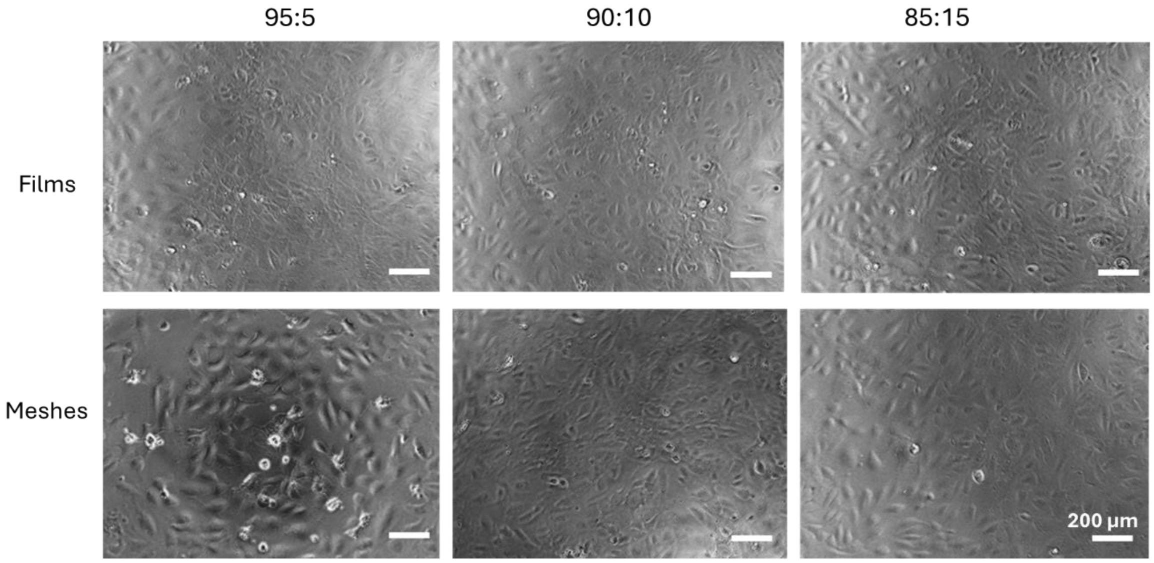


Figure S 7: Brightfield images of HUVECs seeded on films and meshes after 48 hours of cultivation following treatment with extracts.

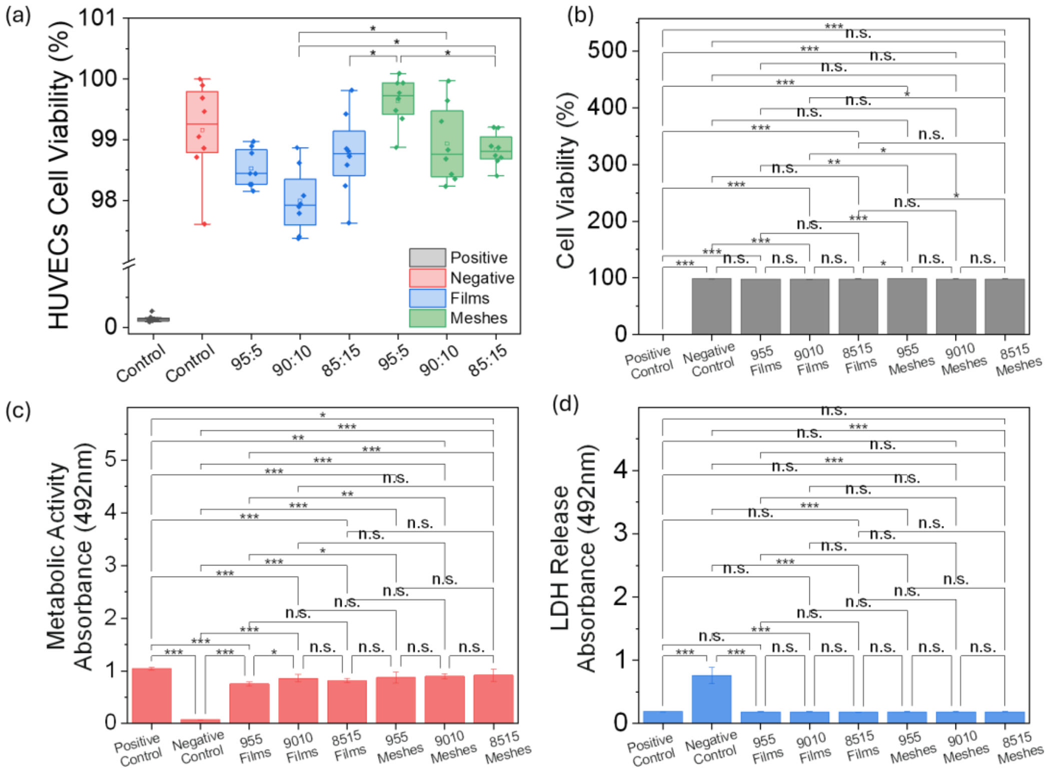


Figure S 8: HUVECs cell culture after 48 hours of cultivation for indirect cytotoxicity assays. (a) Representative data showing HUVECs' viability, and (b-d) corresponding detailed

statistical significant differences (b) cell viability activity; (c) metabolic activity measured by MTS assay; and (d) quantification of LDH release. *p<=0.05; **p<=0.01; ***p<=0.001.

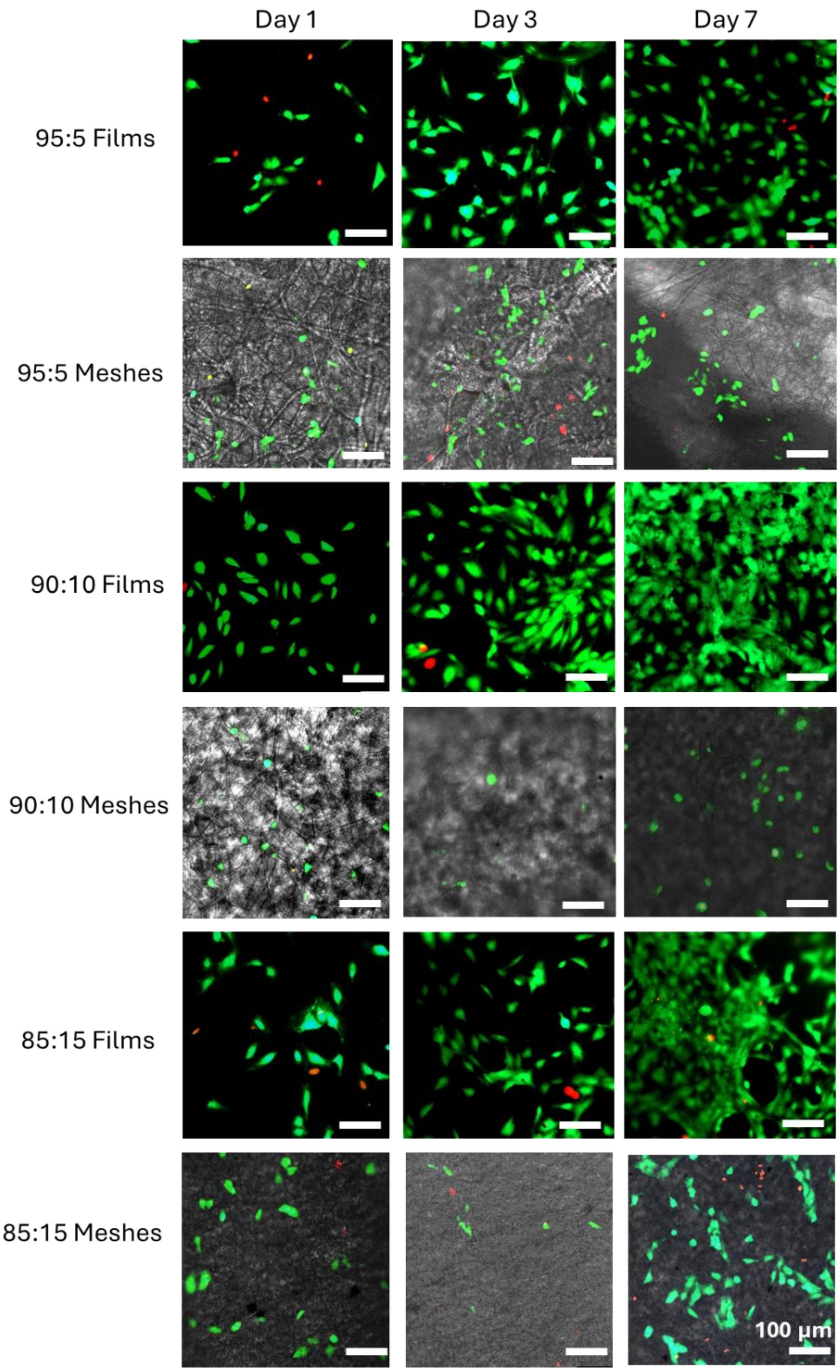


Figure S 9: Fluorescence microscopy images (20X magnification) showing live (green) and dead (red) HUVECs cultured on films and meshes composed of various PLLA-*co*-CL: PDLA blend ratios over 1, 3, and 7 days. Scale bar: 100 µm.

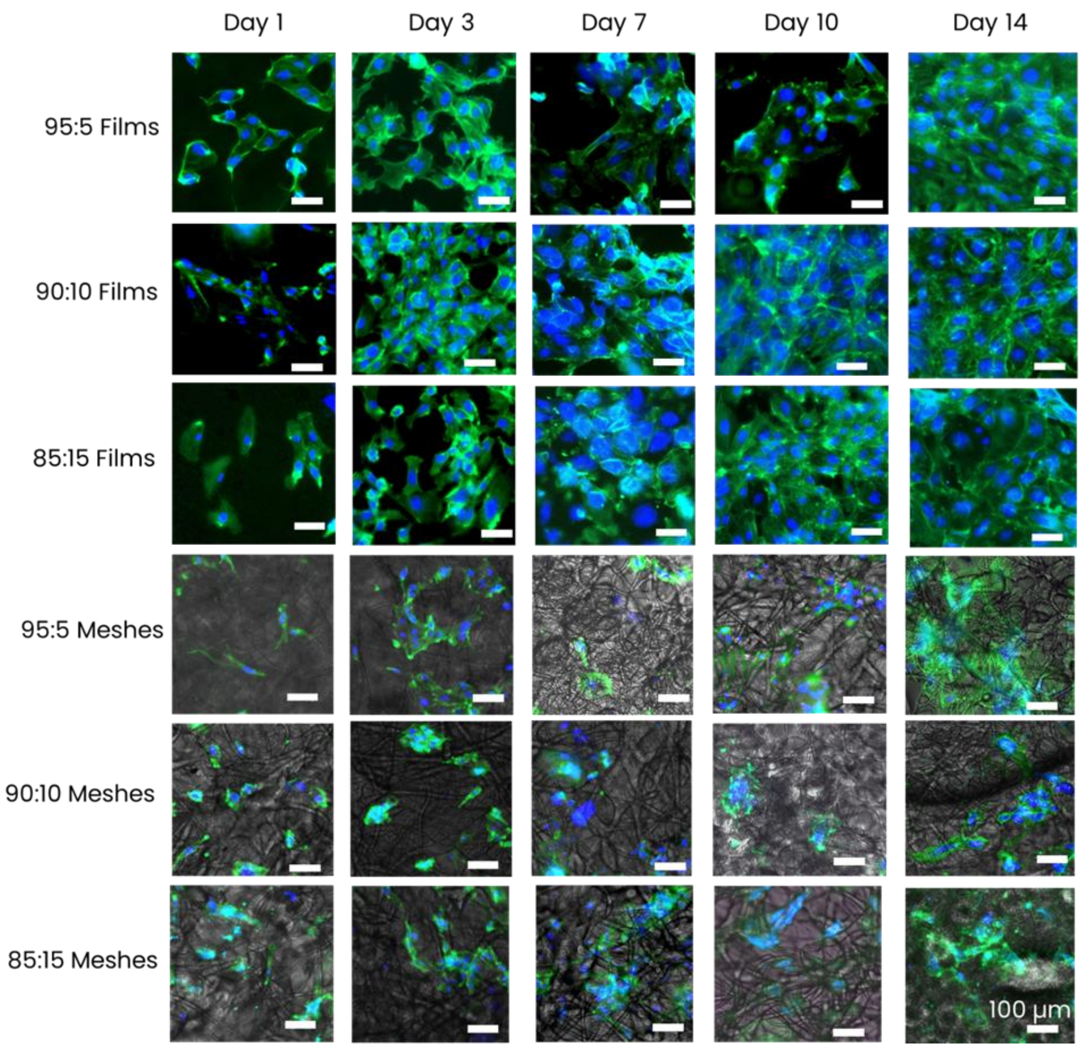


Figure S 10: Fluorescence microscopy images of HUVECs cultured on films and meshes for 1, 3, 7, 10, and 14 days. Actin filaments are stained green, and cell nuclei are stained blue, highlighting cytoskeletal organization and cell distribution. Scale bar: 100 µm.

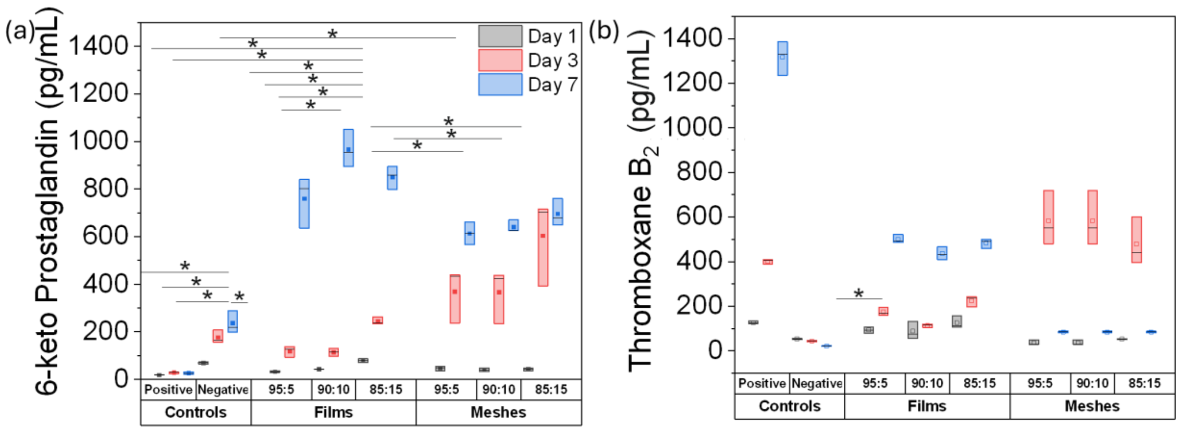


Figure S 11: Secretion of vasoactive substances by HUVECs cultured on scaffolds with a density of 20,000 cells/well for a period of 7 days. The total concentrations of (a) prostacyclin and (b) thromboxane $B_2$, quantified using ELISA. Statistical significance was determined by ANOVA ($p \leq 0.05$), with significant differences indicated by (*).
